# Effects of urban living environments on mental health in adults

**DOI:** 10.1038/s41591-023-02365-w

**Published:** 2023-06-15

**Authors:** Jiayuan Xu, Nana Liu, Elli Polemiti, Liliana Garcia-Mondragon, Jie Tang, Xiaoxuan Liu, Tristram Lett, Le Yu, Markus M. Nöthen, Jianfeng Feng, Chunshui Yu, Andre Marquand, Gunter Schumann, the environMENTAL Consortium, Henrik Walter, Andreas Heinz, Markus Ralser, Sven Twardziok, Nilakshi Vaidya, Emin Serin, Marcel Jentsch, Esther Hitchen, Roland Eils, Ulrike-Helene Taron, Tatjana Schütz, Kerstin Schepanski, Jamie Banks, Tobias Banaschewski, Karina Jansone, Nina Christmann, Andreas Meyer-Lindenberg, Heike Tost, Nathalie Holz, Emanuel Schwarz, Argyris Stringaris, Maja Neidhart, Frauke Nees, Sebastian Siehl, Ole A. Andreassen, Lars T. Westlye, Dennis van der Meer, Sara Fernandez, Rikka Kjelkenes, Helga Ask, Michael Rapp, Mira Tschorn, Sarah Jane Böttger, Gaia Novarino, Lena Marr, Mel Slater, Guillem Feixas Viapiana, Francisco Eiroa Orosa, Jaime Gallego, Alvaro Pastor, Andreas Forstner, Per Hoffmann, Markus M. Nöthen, Andreas J. Forstner, Isabelle Claus, Abbi Miller, Stefanie Heilmann-Heimbach, Peter Sommer, Mona Boye, Johannes Wilbertz, Karen Schmitt, Viktor Jirsa, Spase Petkoski, Séverine Pitel, Lisa Otten, Anastasios-Polykarpos Athanasiadis, Charlie Pearmund, Bernhard Spanlang, Elena Alvarez, Mavi Sanchez, Arantxa Giner, Sören Hese, Paul Renner, Tianye Jia, Yanting Gong, Yunman Xia, Xiao Chang, Vince Calhoun, Jingyu Liu, Paul Thompson, Nicholas Clinton, Sylvane Desrivieres, Allan H. Young, Bernd Stahl, George Ogoh

**Affiliations:** 1 Department of Radiology and Tianjin Key Laboratory of Functional Imaging, Tianjin Medical University General Hospital, Tianjin, People’s Republic of China; 2 Centre for Population Neuroscience and Stratified Medicine, Institute for Science and Technology of Brain-inspired Intelligence, Fudan University, Shanghai, People’s Republic of China; 3 Centre for Population Neuroscience and Stratified Medicine (PONS), Charite Mental Health, Department of Psychiatry and Neurosciences, CCM, Charite Universitätsmedizin Berlin, Berlin, Germany; 4 International Max Planck Research School for Translational Psychiatry, Munich, Germany; 5 Aerospace Information Research Institute, Chinese Academy of Sciences, Beijing, People’s Republic of China; 6 Department of Earth System Science, Ministry of Education Key Laboratory for Earth System Modeling, Institute for Global Change Studies, Tsinghua University, Beijing, People’s Republic of China; 7 Department of Earth System Science, Ministry of Education Ecological Field Station for East Asian Migratory Birds, Tsinghua University, Beijing, People’s Republic of China; 8 Institute of Human Genetics, University Hospital of Bonn, Bonn, Germany; 9 Institute of Science and Technology for Brain-Inspired Intelligence, Fudan University, Shanghai, People’s Republic of China; 10 CAS Center for Excellence in Brain Science and Intelligence Technology, Chinese Academy of Sciences, Shanghai, People’s Republic of China; 11 Predictive Clinical Neuroscience Group at the Donders Institute, Radboud University Medical Center, Nijmegen, the Netherlands; 12 Department of Psychiatry and Psychotherapy, Charite Universitaetsmedizin Berlin, Berlin, Germany; 13 Department of Biochemistry, Charite Universitaetsmedizin Berlin, Berlin, Germany; 14 Nuffield Department of Medicine, University of Oxford, Oxford, UK; 15 Max Planck Institute for Molecular Genetics, Berlin, Germany; 16 Berlin Institute of Health at Charité-Universitätsmedizin Berlin,Center of Digital Health, Berlin, Germany; 17 Institute of Meteorology, Free University Berlin, Berlin, Germany; 18 Department of Child and Adolescent Psychiatry and Psychotherapy, Central Institute of Mental Health, Medical Faculty Mannheim, Heidelberg University, Heidelberg, Germany; 19 Central Institute of Mental Health, Medical Faculty Mannheim, Heidelberg University, Heidelberg, Germany; 20 Donders Institute for Brain, Cognition and Behavior Radboud University Nijmegen, Nijmegen, the Netherlands; 21 Department for Cognitive Neuroscience, Radboud University Medical Center Nijmegen, Nijmegen, the Netherlands; 22 Institute of Medical Psychology and Medical Sociology, University Medical Center Schleswig-Holstein, Kiel University, Kiel, Germany; 23 Institute of Cognitive and Clinical Neuroscience, Central Institute of Mental Health, Medical Faculty Mannheim, Heidelberg University, Mannheim, Germany; 24 Norwegian Centre for Mental Disorders Research (NORMENT), Division of Mental Health and Addiction, Oslo University Hospital & Institute of Clinical Medicine, University of Oslo, Oslo, Norway; 25 K.G. Jebsen Centre for Neurodevelopmental Disorders, University of Oslo, Oslo, Norway; 26 Department of Psychology, University of Oslo, Oslo, Norway; 27 Department of Mental Disorders, Norwegian Institute of Public Health, Oslo, Norway; 28 Department for Social and Preventive Medicine, University of Potsdam, Potsdam, Germany; 29 Institute of Science and Technology, Klosterneuburg, Austria; 30 Event Lab, Department of Clinical Psychology and Psychobiology, Institute of Neurosciences, University of Barcelona, Casanova, Barcelona, Spain; 31 Department of Computer Science, University College London, London, UK; 32 Institut de Neurociències, Universitat de Barcelona, Campus de Mundet, Barcelona, Spain; 33 Institute of Neuroscience and Medicine (INM-1), Research Center Jülich, Jülich, Germany; 34 Institute of Human Genetics, University of Bonn, School of Medicine & University Hospital Bonn, Bonn, Germany; 35 Ksilink, Strasbourg, France; 36 Aix Marseille Université, Institut National de la Santé et de la Recherche Médicale (Inserm), Institut de Neurosciences des Systèmes (INS) UMR1106, Marseille, France; 37 Virtual Bodyworks, Barcelona, Spain; 38 Institute of Geography, Friedrich Schiller University Jena, Jena, Germany; 39 Tri-institutional Center for Translational Research in Neuroimaging and Data Science (TReNDS), Georgia State University/Georgia Institute of Technology, Emory, Atlanta, GA USA; 40 Imaging Genetics Center, Mark & Mary Stevens Institute for Neuroimaging & Informatics, Los Angeles, CA USA; 41 Google, Inc., Mountain View, CA USA; 42 Social, Genetic and Developmental Psychiatry Centre, Institute of Psychiatry, Psychology & Neuroscience, King’s College London, London, UK; 43 Institute of Psychiatry, Psychology & Neuroscience, King’s College London, London, UK; 44 School of Computer Science, University of Nottingham, Nottingham, UK

**Keywords:** Risk factors, Genome-wide association studies

## Abstract

Urban-living individuals are exposed to many environmental factors that may combine and interact to influence mental health. While individual factors of an urban environment have been investigated in isolation, no attempt has been made to model how complex, real-life exposure to living in the city relates to brain and mental health, and how this is moderated by genetic factors. Using the data of 156,075 participants from the UK Biobank, we carried out sparse canonical correlation analyses to investigate the relationships between urban environments and psychiatric symptoms. We found an environmental profile of social deprivation, air pollution, street network and urban land-use density that was positively correlated with an affective symptom group (*r* = 0.22, *P*_perm_ < 0.001), mediated by brain volume differences consistent with reward processing, and moderated by genes enriched for stress response, including *CRHR1*, explaining 2.01% of the variance in brain volume differences. Protective factors such as greenness and generous destination accessibility were negatively correlated with an anxiety symptom group (*r* = 0.10, *P*_perm_ < 0.001), mediated by brain regions necessary for emotion regulation and moderated by *EXD3*, explaining 1.65% of the variance. The third urban environmental profile was correlated with an emotional instability symptom group (*r* = 0.03, *P*_perm_ < 0.001). Our findings suggest that different environmental profiles of urban living may influence specific psychiatric symptom groups through distinct neurobiological pathways.

## Abstract (web summary)

Analyses of data from the UK Biobank reveal different urban living environments that are associated with affective, anxiety and emotional instability symptom groups and mediated by distinct neurological and genetic pathways in adults.

## Main

More than 50% of the world population lives in urban areas; by 2050, two-thirds will live in cities^1^. Thus, environments are going through drastic transformations: living in urban areas is characterized by higher-density residential and commercial buildings^1^, concomitant reduced access to green areas^2^, increased exposure to potentially licit and illicit substance use^3^ and more stressful social conditions^4^. At the same time, urban residents potentially benefit from better infrastructure and more work opportunities than residents residing in rural areas^1^.

The impact of the urban living environment on mental health is not well understood. Physical health is thought to be better in urban areas compared to rural areas^5^. However, there is evidence that adult individuals in urban environments are at higher risk of experiencing mental health conditions^6^, although results are contradicting^7^. While there has been a focus on the link between urbanicity and schizophrenia^8^, the most prevalent mental health conditions linked to urbanicity are symptoms of depression and anxiety^6,9–11^.

While previous studies investigated isolated environmental factors relevant to urban living, such as green spaces^12^ and socioeconomic deprivation^13^, these isolated factors have not been considered in the wider environmental context that characterizes a living environment. To develop targeted prevention and intervention programs ranging from urban planning to individual psychosocial programs, it is neither sufficient to regard urbanicity as one general risk factor nor to focus on single isolated environmental factors alone. The urban environment, as any other living environment, consists of simultaneous interacting factors, which may form profiles that together can reduce or increase the risk of psychiatric disorders.

The relationships between psychiatric disorders and brain structure with exposure to environmental profiles are currently unknown, either in urban or other settings. Furthermore, exposure to environmental adversity does not result in a uniform response but shows individual differences^14^, of which genetic variations are one important source^15^. Activity of biological pathways, such as stress response, that are mediators of the effects of stressful environmental stimuli on brain and psychiatric disorders vary depending on genotypes^16^.

**Fig. 1:**
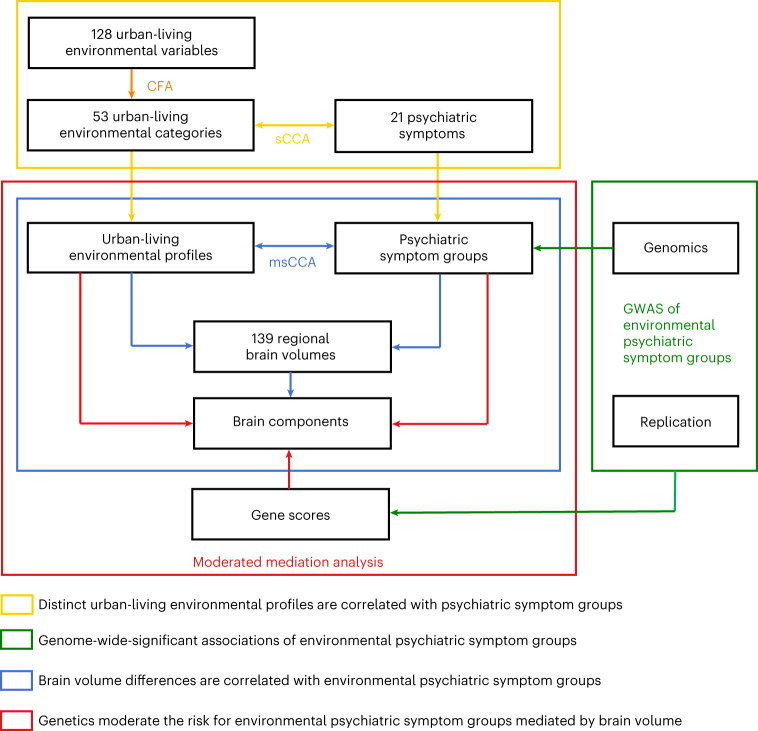
Characterization of the study design. In 141,087 UKB-non-NI participants, we identified urban environmental profiles correlated to psychiatric symptom groups using sCCA with a train–test dataset split design. Next, we carried out GWAS analyses of the symptom groups in 76,508 participants with complete genomic, urban environmental categories and psychiatric symptoms from the UKB-non-NI dataset. The UKB-NI dataset data (*n* = 14,988) was used for independent replication of the multivariate relationship between urban environmental profiles, genes and symptom groups, and for additional neuroimaging analyses. We analyzed the relationships between urban environmental profiles, regional brain volume and symptom groups using msCCA with a train–test dataset split design. Using a moderated mediation analysis, we then investigated the interaction effect between urban environmental profiles and genetics on psychiatric symptoms groups mediated by brain components in 8,705 participants with complete genomic, urban environmental categories, regional brain volume and symptoms of mental illness in the UKB-NI dataset.

**Extended Data Fig. 1:**
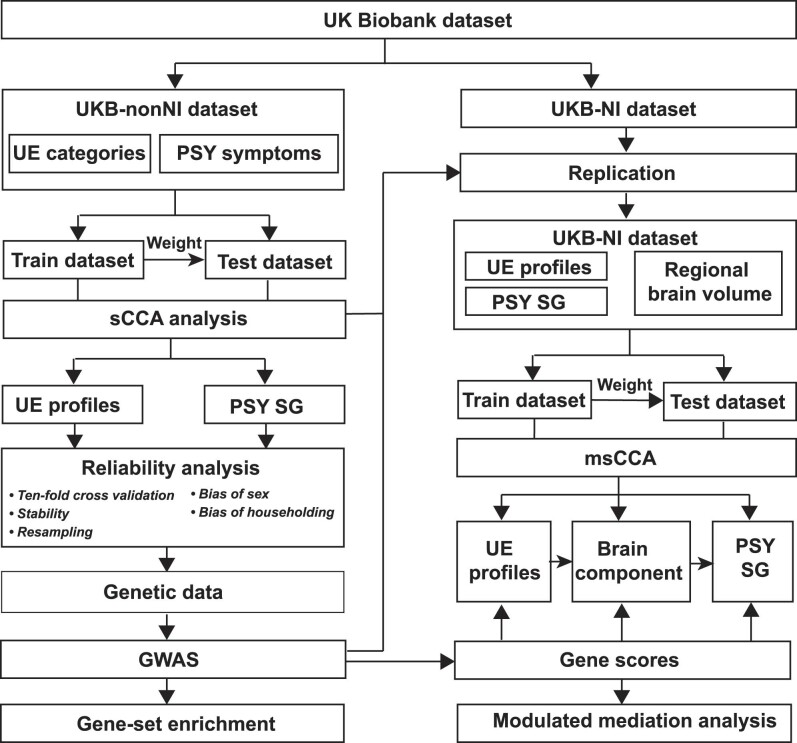
A schematic summary of the study design. GWAS, genome-wide association analysis; msCCA, multiple sparse canonical-correlation analysis; PSY, psychiatric; sCCA, sparse canonical-correlation analysis; SG, symptom groups; UE, urban-environmental; UKB-non-NI dataset and UKB-NI dataset, participants from UK Biobank with complete urban-living environmental data and psychiatric symptoms (n = 156,075) were divided into datasets without neuroimaging data (UKB-non-NI dataset, n = 141,087) and with neuroimaging data (UKB-NI dataset, n = 14,988).

In this study, we aim to capture the complexity of the urban living environment by combining measures of physical environment with socioeconomic data. We identify urban living environmental profiles and relate them to psychiatric symptom groups. We aim to understand what combinations of environmental factors are most relevant for these psychiatric symptoms, and how within these combinations each single factor contributes to the risk or resilience of mental health symptoms. We also identify regional brain areas that mediate the effect of these different environmental profiles on psychiatric symptom groups. We investigate genetic variations derived from genome-wide analyses of these psychiatric symptom groups and test them for moderation of the regional brain volumes correlated to urban environmental profiles (Fig. 1 and Extended Data Fig. 1).

## Results

**Extended Data Fig. 2:**
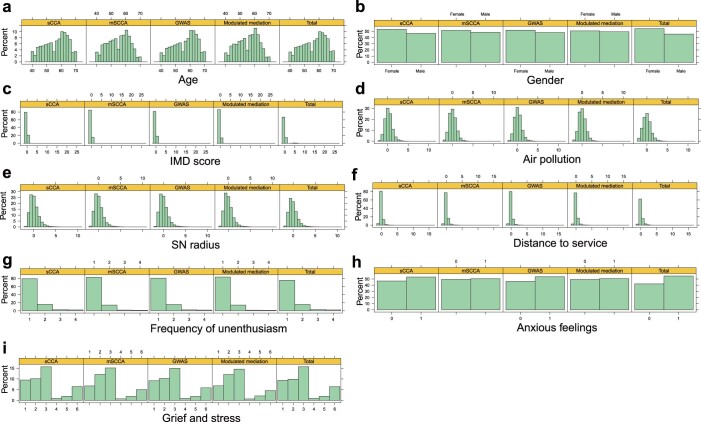
Histograms of demographic, urban-living environment category and psychiatric symptoms variables in the analytical sample and total sample in UK Biobank. Histograms distributions of demographic variables of age (**a**) and gender (**b**), urban-living environment category variables with top weight including IMD score (**c**), air pollution (**d**), street network radius (**e**) and distance to service (**f**), psychiatric symptoms variables with top weight including frequency of unenthusiasm (**g**), anxious feelings (**h**), grief and stress (**i**) in the analytical sample and total sample in UK Biobank.

Our analyses were carried out in a subset of 156,075 adult participants from the UK Biobank (UKB), aged 41–77 years (mean age: 59.11 years) and living predominantly in urban areas. This subset was assessed for 128 urban living environmental variables linked to their home address (Supplementary Tables 1–3) and 21 psychiatric symptoms (Supplementary Table 4). The urban living environmental areas included air and sound pollution, traffic, green space proximity, coastal proximity, water proximity, socioeconomic indices of multiple deprivation (IMD), building class, distance to destinations (for example, GP practice, post office), land use density (LD), terrain, normalized difference vegetation index (NDVI) (a measure of greenness), and street network (SN) accessibility (Supplementary Tables 1–3). Participants from the UKB with complete urban living environmental variables and psychiatric symptom data were divided into datasets without neuroimaging data (UKB-non-NI) (*n* = 141,087) and with neuroimaging data (UKB-NI) (*n* = 14,988). At the time of our analyses, brain neuroimaging was ascertained in 42,796 participants, of which 14,988 had complete neuroimaging, urban-living environmental and psychiatric symptom assessments. Schematic summaries are shown in Fig. 1 and Extended Data Fig. 1. Demographic information on the specific statistical analysis is shown in Table 1. Distribution of demographic variables in each statistical subsets showed that potential attrition bias was evident for some variables (for example, sex), whereas most other variables (for example, age) were highly similar across the different subsets, suggesting that the magnitude of bias was small (Extended Data Fig. 2).

**Table 1:** Demographics of UKB participants used in the specific statistical analyses

Dataset	Statistical analysis	Required data	Sample size	Age range, years	Age statistics^a^	Sex (M/F)
**Total sample**	**–**	**–**	156,075	41–77	59.11 (8.09)	72,770/83,305
**UKB-non-NI**	**CFA and sCCA**	Urban living environment, mental health	141,087	41–77	59.25 (8.14)	65,505/75,582
**UKB-non-NI**	**GWAS**	Genomic, urban living environment, mental health	76,508	41–75	59.55 (8.02)	36,557/39,951
**UKB-NI**	**msCCA**	Urban living environment, neuroimaging, mental health	14,988	41–74	57.77 (7.49)	7,265/7,723
**UKB-NI**	**Modulated mediation**	Genomic, urban living environment, neuroimaging, mental health	8,705	41–74	58.06 (7.42)	4,278/4,427

^a^Age statistics are shown as the mean and s.d. UKB-non-NI and UKB-NI datasets: participants from the UKB with complete urban living environmental data and mental health data (*n* = 156,075) were divided into UKB-non-NI and UKB-NI datasets.

### Correlation of urban living environmental profiles with psychiatric symptom groups

**Fig. 2:**
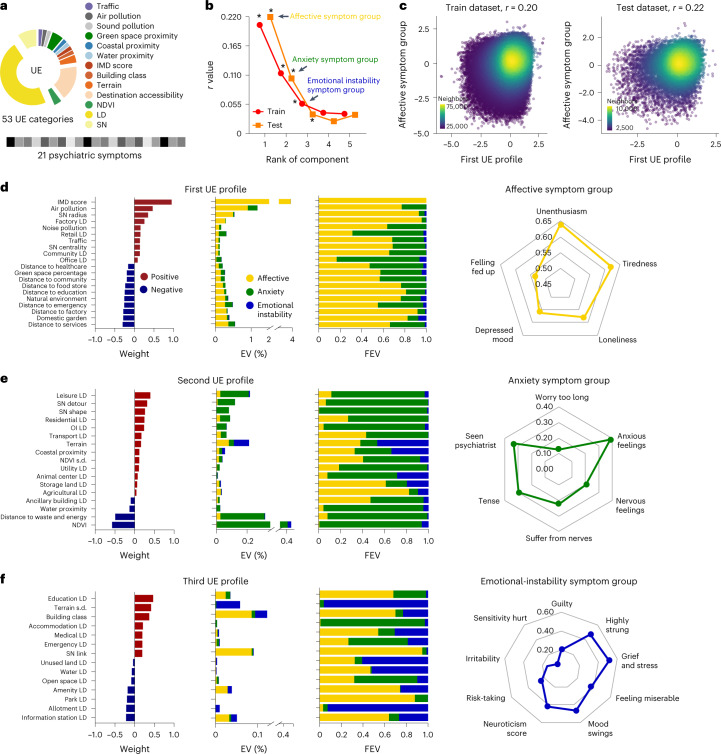
Distinct urban environmental profiles are correlated with specific psychiatric symptom groups. **a**, Fifty-three urban environmental categories belonging to 13 areas (the dots on the right) and 21 psychiatric symptoms are included. UE, urban living environment. **b**, The sCCA model linking 53 urban environmental categories to 21 psychiatric symptoms identified three significant canonical correlates in the training datasets (red dot), including affective symptom (*r* = 0.20, *P*_perm_ < 0.001), anxiety symptom (*r* = 0.11, *P*_perm_ < 0.001) and emotional instability symptom (*r* = 0.05, *P*_perm_ < 0.001) groups. These results remained significant in the test datasets of affective (*r* = 0.22, *P*_perm_ < 0.001, *P*_FDR_ < 0.001), anxiety (*r* = 0.10, *P*_perm_ < 0.001, *P*_FDR_ < 0.001) and emotional instability (*r* = 0.03, *P*_perm_ < 0.001, *P*_FDR_ < 0.001) (orange square) symptom groups. *P* values were estimated using one-sided *P*_perm_ with FDR correction for multiple comparisons (*P*_FDR_). **c**, A correlation map between the first urban living environmental profile and affective symptom group in the training (left) and test (right) datasets. **d–f**, In the first (**d**), second (**e**) and third (**f**) correlates, urban environmental categories contributing to this profile are shown in the first column. EV and fraction of EV of crossloadings of each urban environmental category on each of the three symptom groups are shown in the second and third columns. Symptoms of mental illness contributing to this group are shown on the right radar plots. The affective, anxiety and emotional instability symptom groups are shown in yellow, green and blue. OI, object of interest.

**Extended Data Fig. 3:**
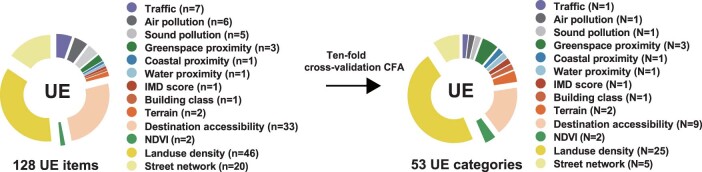
Urban-environmental categories construction. 53 urban-living environment categories composed of 128 variables were included in the study. Among these, 34 categories had one independent environmental variable. In the remaining categories, redundancy between related environmental variables was avoided by collapsing the information into 19 latent environmental categories using ten-fold cross-validation confirmatory-factor-analysis (CFA).

Fifty-three urban living environmental categories consisting of 128 variables were included in the study (Fig. 2a and Extended Data Fig. 3). Among these, 34 categories had one independent environmental variable. In the remaining categories, redundancy between related environmental variables was avoided by collapsing the information into 19 latent environmental categories using tenfold cross-validation confirmatory factor analysis (CFA) (Methods and Extended Data Fig. 3). To investigate the relationship between urban living environment and psychiatric symptoms, we used sparse canonical correlation analyses (sCCA) to link these 53 independent urban living environment categories with 21 psychiatric symptoms (Methods). To enhance the stability of the sCCA, we resampled the data and retained only variables above 90% across the resample^17^ (Methods). To avoid overestimating the variance shared between urban living environment categories and psychiatric symptoms, we used a split-data analysis design with a training dataset of 90% of the data (*n* = 126,978), and a test dataset of 10% (*n* = 14,109) in the 141,087 participants of the UKB-non-NI dataset.

#### Affective symptom group

We found a significant relationship between an urban living environmental profile and a group consisting of five psychiatric symptoms in the training dataset (*r* = 0.20, *P*_perm_ < 0.001, explained variance (EV) = 4.09%), which was replicated in the test dataset (*r* = 0.22, *P*_perm_ < 0.001, *P*_FDR_ < 0.001, EV = 4.71%) (Fig. 2b,c). This psychiatric symptom group consisted of frequency of unenthusiasm, frequency of tiredness, loneliness, frequency of depressed mood and feeling fed-up (Fig. 2d), which we summarized as the affective symptom group. The affective symptom group was positively correlated with environmental factors that included an IMD score, air and sound pollution, measures of SN accessibility (street radial and centrality), traffic and density of urban infrastructures (factories, retail, offices and community). This group was negatively correlated with distance to urban facilities (services, factories, emergency, education, food stores, community and healthcare) and green space proximity (percentage of domestic garden, natural environment and green space) (Fig. 2d and Supplementary Table 5).

**Extended Data Fig. 4:**
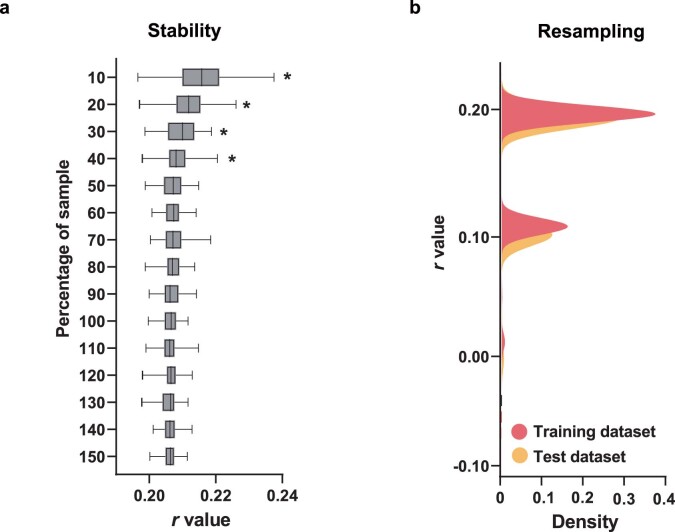
Robustness assessment. **a**. We used bootstrapping to resample the training data (with replacement) 1000 times, each containing 10% to 150% of the training dataset in 10% increments. Box and whiskers graphs showed the correlation coefficient *r* value in each resampling. The bounds of box demonstrated data extend from the 25th to 75th percentiles. The centre line in the box was plotted at the median. The whiskers went down to the smallest and up to the largest value. Stability in correlation coefficient after about 40% of the sample size were observed. **b**. To estimate the stability of the findings across subsamples, we resampled the same proportion 90% of original sample size as train dataset for 1000 times, reran the sCCA algorithm and calculated the correlation between the resulting feature in the remaining 10% test dataset.

**Extended Data Fig. 5:**
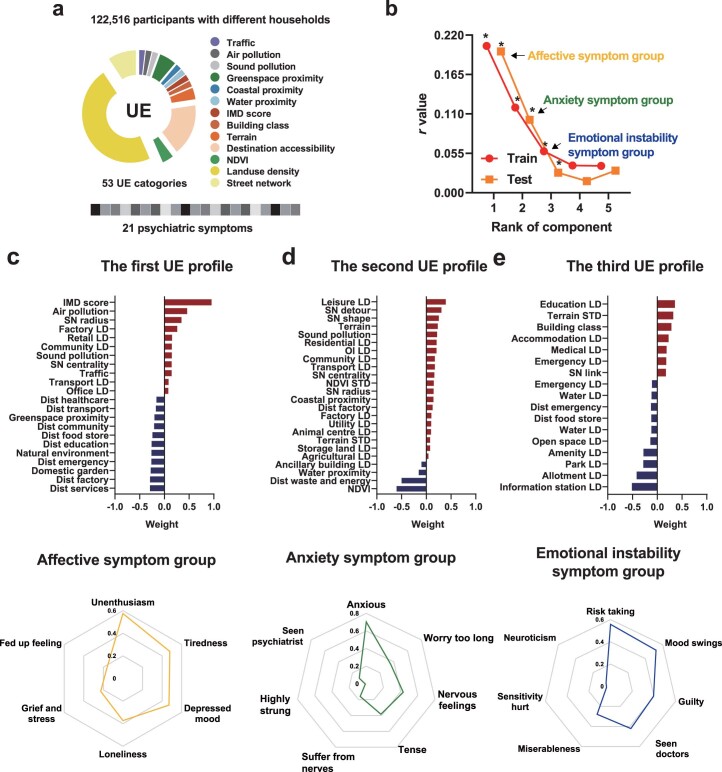
The sCCA-regression between urban-living environment categories and psychiatric symptoms in 122,516 participants with different households. **a**. Top left: A total of 53 categories of urban-living environment belonged to 13 areas are included. Top right: Each dot demonstrates the 13 areas of urban-living environment. Bottom: A total of 21 psychiatric symptoms are included; **b**. The sCCA-regression model linking 53 urban-living environment categories to 21 psychiatric symptoms identified three significant correlates in train datasets (red dot), including affective symptoms group (*r* = 0.20, *P*_*perm*_ < 0.001), anxiety symptoms group (*r* = 0.12, *P*_*perm*_ < 0.001) and emotional instability symptoms group (*r* = 0.06, *P*_*perm*_ < 0.001). These results were still significant in test datasets of affective (*r* = 0.20, *P*_*perm*_ < 0.001, *P*_*FDR*_ < 0.001), anxiety (*r* = 0.10, *P*_*perm*_ < 0.001, *P*_*FDR*_ < 0.001) and emotional instability symptom-groups (*r* = 0.03, *P*_*perm*_ < 0.001, *P*_*FDR*_ < 0.001) (orange square). *P* values here were estimated using two-sided permutation tests (*P*_*perm*_) and FDR correction have been made for multiple comparisons (*P*_*FDR*_); **c**–**e**. In the first (**c**), second (**d**) and third (**e**) correlates, urban-living environment profiles contributing to this relationship were shown on the top, psychiatric symptoms contributing to this relationship were shown on the bottom radar plots. Dist., Distance; LD, landuse density; IMD, Index of Multiple Deprivation; NDVI, normalized difference vegetation index; SG, symptoms of group; SN, street network; STD, standard deviation; UE, urban-living environment.

Internal validations using bootstrapping and resampling (Methods) confirmed the associations between urban living environmental profiles and affective symptom group (Extended Data Fig. 4 and Supplementary Table 6). To avoid overfitting, we repeated the sCCA regression using tenfold cross-validation, yielding highly similar accuracy (Supplementary Table 7). No specific sex effect was detected (Supplementary Table 8). To rule out the influence of genetically unrelated participants in the same household, we repeated the sCCA regression and confirmed the results in the 122,516 participants from different households using the same split-data design (Methods and Extended Data Fig. 5).

Our results indicate that the affective symptom group was positively correlated with an environment profile dominated by high levels of social deprivation and air pollution, and to a lesser extent SN, traffic and short distance to infrastructure facilities. Other factors of urbanicity, such as various forms of green space and social infrastructure, are protective.

#### Anxiety symptom group

**Extended Data Fig. 6:**
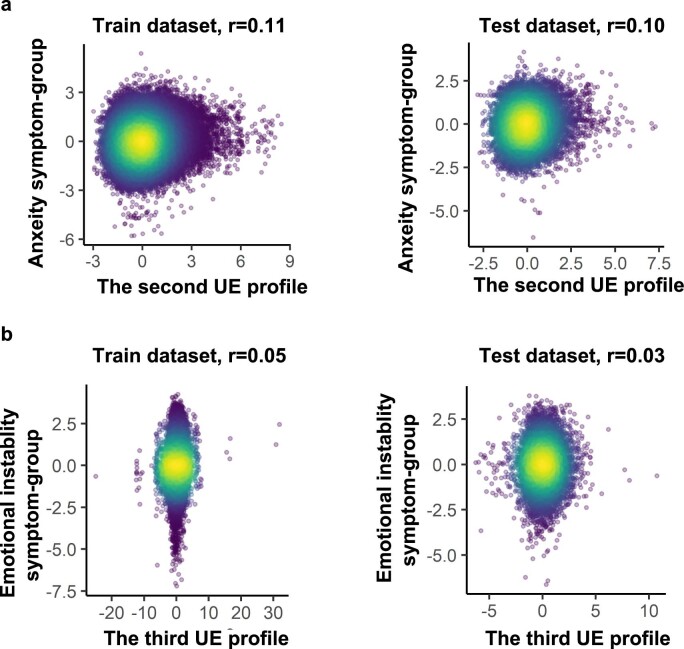
Correlations between urban-living environmental profile and psychiatric symptom groups. Correlation maps between the second urban-living environmental profile and anxiety symptom-group (**a**) as well as the third urban-living environmental profile and emotional instability symptom-group (**b**).

We identified another psychiatric symptom group after projection deflation^17,18^ (Methods), which consists of anxious feelings, seeing a psychiatrist, feeling tense, suffering from nerves, nervous feelings and worrying too long (Fig. 2e), grouped together as the anxiety symptom group. The anxiety symptom group was significantly associated with the second urban environmental profile in the training (*r* = 0.11, *P*_perm_ < 0.001) and test (*r* = 0.10, *P*_perm_ < 0.001, *P*_FDR_ < 0.001, EV = 1.03%) datasets (Fig. 2b and Extended Data Fig. 6). This symptom group was positively correlated with dense urban buildup, including density of leisure places, SN detour and shape, mean terrain, coast proximity, variation of NDVI and density of mixed urban infrastructure (residential, transport, utility, animal center, storage land and agriculture), while being negatively correlated with mean NDVI, distance to waste and energy, and water proximity (Fig. 2e and Supplementary Table 5). Thus, the second urban living environmental profile captured a different profile defined by generous land use and proximity to nature, features that are protective against symptoms of anxiety.

#### Emotional instability symptom group

We identified a third group of psychiatric symptoms that consisted of frequency of feeling highly strung, feeling miserable, mood swings, neuroticism score (Supplementary Table 4), risk-taking, irritability and sensitivity, hurt feelings, grief and stress (Fig. 2f), which we termed the emotional instability symptom group. The emotional instability symptom group was positively correlated with the density of education facilities, variation of terrain, building class (flats in high-rise buildings, terraced houses), SN link characteristics, density of accommodation, and medical and emergency facilities, while being negatively correlated with density of unused land, density of water, open space, amenities, park, allotment and information stations, as well as distance to a food store (training dataset: *r* = 0.05, *P*_perm_ < 0.001; test dataset: *r* = 0.03, *P*_perm_ < 0.001, *P*_FDR_ < 0.001; Fig. 2f, Extended Data Fig. 6 and Supplementary Table 5).

#### Replication and pleiotropy analyses

We independently replicated these correlations in the UKB dataset with neuroimaging (UBB-NI) (*n* = 14,988) by applying the same sCCA split design (90%/10%). The replication analysis yielded three statistically significant correlations between environmental profiles and psychiatric symptom groups in the training and test datasets, which were identical to those of the primary analyses. In the affective symptom group, the canonical correlation *r* value was 0.17 in the training dataset (*n* = 13,490; *P*_perm_ < 0.001) and 0.10 in the test dataset (*n* = 1,498; *P*_perm_ < 0.001, *P*_FDR_ < 0.001). In the anxiety symptom group, the canonical correlation *r* value was 0.11 in the training dataset (*P*_perm_ < 0.001) and 0.03 in the test dataset (*P*_perm_ < 0.001, *P*_FDR_ < 0.001). In the emotional instability symptom group, the canonical correlation was 0.10 in the training dataset (*P*_perm_ < 0.001) and 0.02 in the test dataset (*P*_perm_ = 0.004, *P*_FDR_ = 0.027).

Environmental factors may be pleiotropic, that is, risk factors for more than one psychiatric symptom group. We tested pleiotropy by measuring the contribution of each urban living environmental factor to the correlation across and within different psychiatric symptom groups using a non-sCCA regression^19^. The result of this analysis is expressed as the fraction of explained variance (FEV). The FEV of the affective symptom group by the factors of the first urban environmental profile was 68.43%; the second environmental profile was 29.34%; and the third environmental profile was 2.22% (Fig. 2d). The FEV of anxiety symptoms by the second environmental profile was 64.24%; the first environmental profile was 25.62%; and the third environmental profile was 10.12% (Fig. 2e). In the case of the emotional instability symptom group, we found substantial pleiotropy: the FEV of the emotional instability symptom group by the factors of the third environmental profile was 31.58%; the first environmental profile was 46.76%; and the second environmental profile was 21.65% (Fig. 2f). This high degree of pleiotropy can most probably be accounted for by how sCCA estimates successive components using the projection deflation approach^17,18^. The proportion of covariance explained by the third emotional instability symptom group was smaller than that explained by the affective and anxiety symptom groups, which may have produced a low *r* value between the third urban living environmental profile and emotional instability symptom group.

### Genome-wide-significant associations with environmental psychiatric symptom groups

We performed genome-wide association study (GWAS) analyses of the canonical covariates of the affective, anxiety and emotional instability symptom groups in 76,508 participants with complete genetic, urban environment and psychiatric symptoms in the UKB-non-NI datasets (Table 1). Gene set enrichment analysis (GSEA) using ToppGene^20^ was performed to explore the biological mechanisms underlying genes associated with the psychiatric symptom groups. To reduce dimensionality, we generated scores for individual genes where significant single-nucleotide polymorphisms (SNPs) were localized (Methods). The individual gene scores were calculated as the sum of the count of risk alleles multiplied by the corresponding *β* value from the GWAS across the index SNPs of each clump after adjusting for linkage disequilibrium (Methods). These gene scores were then analyzed for moderation of the relationship of urban living environmental profile, regional brain volume and psychiatric symptom groups (Fig. 1 and Extended Data Fig. 1).

#### Affective symptom group

**Fig. 3:**
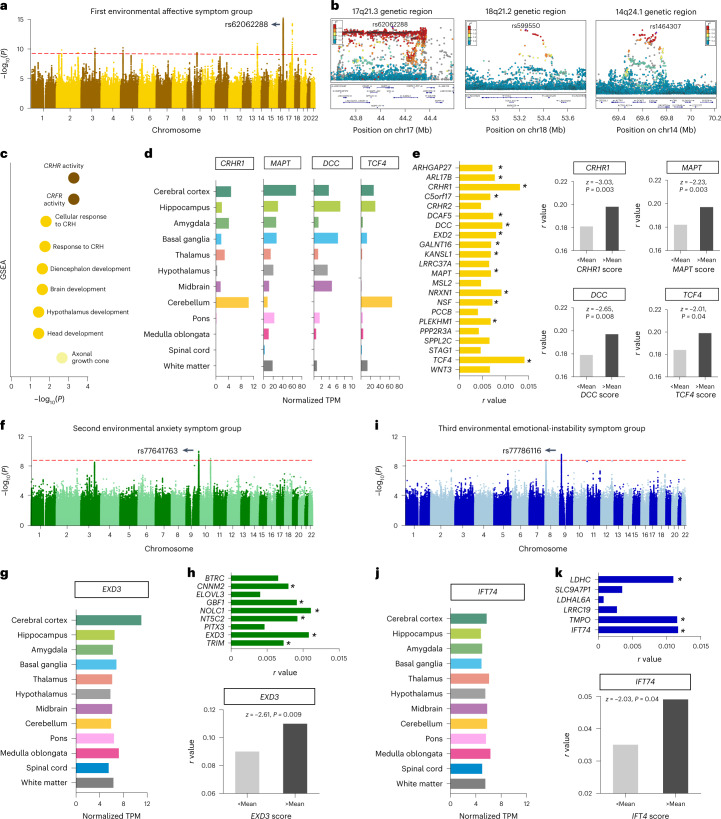
Genome-wide significant associations of environmental psychiatric symptom groups. **a**, The GWAS of the affective symptom group identified 3,436 significantly associated SNPs after Bonferroni correction *P* < 0.05. The lead SNP rs62062288 is located in intron 6 of the *MAPT* gene on chromosome 17q21.3 (two-sided *P* = 6.09 × 10^−15^). **b**, Locus zoom plots of 17q21.3 (left), 18q21.2 (middle) and 14q24.1 (right) in the GWAS analysis of the affective symptom group. The purple dots show the lead SNPs of each genomic region. **c**, GSEA of the affective symptom group-associated 22 genes revealed over-representation in molecular function (dark yellow) of CRH/CRF receptor activity (*Q* = 5.23 × 10^−4^), biological function (yellow) of cellular response to CRH stimulus (*Q* = 0.02) and cellular components (light yellow) in the axonal growth cone (*Q* = 0.002) after Bonferroni correction *P* < 0.05. **d**, *CRHR1*, *MAPT*, *DCC* and *TCF4* gene normalized expression values in 12 brain regions (Human Protein Atlas). **e**, Left, Correlation *r* value between 22 gene scores and the affective symptom group in the replication UKB-NI dataset. The replicated 14 genes with two-sided *P* < 0.05 are marked with an asterisk. Right, Participants with lower *CRHR1* scores (upper left), *MAPT* scores (upper right), *DCC* scores (lower left) and *TCF4* scores (lower right) showed statistically smaller correlations of the first urban environmental profile with the affective symptom group compared to those with higher ones (two-sided *P* < 0.05). **f**, The GWAS of the anxiety symptom group identified 29 significantly associated SNPs after Bonferroni correction *P* < 0.05. The lead SNP rs77641763 is located in intron 15 of the *EXD3* gene of chromosome 9 (two-sided *P* = 9.53 × 10^−11^). **g**, *EXD3* gene normalized expression values in 12 brain regions. **h**, Top, Of the nine genes scores, six gene scores with two-sided *P* < 0.05 (marked with an asterisk) repeatedly correlated with the anxiety symptom group. Bottom, Participants with lower *EXD3* scores showed a statistically smaller correlation of the second urban environmental profile with the anxiety symptom group compared to higher ones (two-sided *P* < 0.05). **i**, The GWAS of the emotional instability symptom group identified ten significantly associated SNPs after Bonferroni correction *P* < 0.05. The lead SNP rs77786116 is located in chromosome 9 of the *IFT74* gene (two-sided *P* = 4.16 × 10^−10^). **j**, *IFT74* gene normalized expression value in 12 brain regions. **k**, Top, Replicated correlations between three gene scores with two-sided *P* < 0.05 (marked with an asterisk) and emotional instability symptom group from six genes. Bottom, Participants with lower *IFT74* scores showed a statistically smaller correlation of the third urban environmental profile with the emotional instability symptom group compared to those with higher scores (two-sided *P* < 0.05). TPM, transcripts per million.

For the affective symptom group, we found 3,436 significant associations with SNPs after Bonferroni correction *P* < 0.05, located in 22 protein-coding genes (Fig. 3a and Supplementary Table 9). The strongest association with the affective symptom group were observed for SNPs localized in a human supergene candidate on chromosome 17q21.3 (Fig. 3b) that encodes several genes previously implicated in psychiatric disorders^21^. The lead SNP was rs62062288, located in intron 6 of the *MAPT* gene of chromosome 17q21.3 (*P* = 6.09 × 10^−15^) (Fig. 3a), a gene that encodes Tau protein in neurons and is involved in affective symptoms^22^. In the same region of chromosome 17q21.3, we found strong association of the affective symptom group with *CRHR1*, a critical regulatory gene for neuroendocrinological and behavioral stress responses^23^. The remaining top associated genes were also encoded in this region, including *ARL17B*, *KANSL1* and *WNT3*. Additional associations were found on chromosome 18q21.2 at the *DCC* and *TCF4* gene locus (Fig. 3b), chromosome 14q24.1 (*DCAF5*, *EXD2* and *GALNT16*) (Fig. 3b), and chromosome 3q22.3 (*STAG1*, *PPP2R3A*, *MSL2* and *PCCB*). In the GSEA of the 22 genes associated with the affective symptom group, we found over-representation in the molecular function of CRH/CRF receptor activity (Bonferroni-corrected *Q* = 5.23 × 10^−4^), most significantly in the biological function of cellular response to CRH stimulus (Bonferroni-corrected *Q* = 0.02) and in the cellular component of the axonal growth cone (Bonferroni-corrected *Q* = 0.002) (Fig. 3c and Supplementary Table 12). All genes were highly expressed in different brain regions in Human Protein Atlas (Fig. 3d). Applying the 22 gene scores, we found statistically different associations between urban living environmental profile and affective symptom group. For example, participants with lower *CRHR1* gene scores showed smaller correlation of the urban living environmental profile with the affective symptom group compared to those with higher *CRHR1* gene scores (*z* = -3.03, *P* = 0.003) (Fig. 3e).

#### Anxiety symptom group

We found significant associations of the anxiety symptom group with 29 SNPs covering 9 genes after Bonferroni correction *P* < 0.05 (Fig. 3f and Supplementary Table 10). The drop in genome-wide-significant hits compared to the affective symptom group GWAS is probably caused by decreased covariance of the anxiety symptom group, after deflation of the correlates of the affective symptom group. The lead SNP in the GWAS of the anxiety symptom group was rs77641763, which is located in intron 15 of the *EXD3* gene of chromosome 9 (*P* = 9.53 × 10^−11^) (Fig. 3f,g). The rs77641763 was associated with suicidal thoughts and behaviors^24^. The other top significant genes include *CNNM2*, *GBF1*, *NOLC1*, *NT5C2* and *TRIM* (Fig. 3h). The nine genes associated with the anxiety symptom group were enriched for small nucleolar ribonucleoprotein complex binding involved in serotonin metabolic processes (Supplementary Table 12). Participants with lower *EXD3* gene scores showed smaller correlation of the urban environmental profile with the anxiety symptom group compared to those with higher *EXD3* gene scores (*z* = −2.61, *P* = 0.009) (Fig. 3h).

#### Emotional instability symptom group

We found significant associations of the emotional instability symptom group with ten SNPs after Bonferroni correction *P* < 0.05 (Fig. 3i and Supplementary Table 11). The lead SNP was rs77786116, which is located in the *IFT74* gene of chromosome 9 (*P* = 4.16 × 10^−10^) (Fig. 3i). *ITF74* is a critical factor in neuronal migration, which is highly expressed in the brain (Fig. 3j) and associated with paranoid schizophrenia^25^. The other top significant genes include *LDHC*, *SLC9A7P1* and *TMPO* (Fig. 3k). Together, they were enriched for cerebellar development processes (Supplementary Table 12). Participants with lower *IFT4* gene scores showed smaller correlation of urban environmental profiles with the emotional instability symptom group compared to those with higher *IFT4* gene scores (*z* = −2.03, *P* = 0.04) (Fig. 3k).

#### Replication

We independently replicated the SNPs significantly associated with the psychiatric symptom groups derived from the discovery GWAS (UKB-non-NI dataset) in 8,705 participants of the independent UKB-NI dataset. The significance threshold was Bonferroni-corrected *P* < 0.05. Of the 3,475 significant SNPs, we replicated 2,034 SNPs associated with the affective symptom group; 18 SNPs were associated with the anxiety symptom group and 3 SNPs were associated with the emotional instability symptom group. We then calculated the corresponding gene scores as before and validated the associations between gene scores and psychiatric symptom groups in the UKB-NI dataset (Supplementary Table 13). Of the 22 gene scores associated with the affective symptom group in the discovery dataset, we replicated 14 genes in the replication analysis of the UKB-NI dataset, including *ARHGAP27*, *ARL17B*, *C5orf17* (*LINC02899*), *CRHR1*, *DCAF5*, *DCC*, *EXD2*, *GALNT16*, *KANSL1*, *MAPT*, *NRXN1*, *NSF*, *PLEKHM1* and *TCF4* (Fig. 3e). Of the 11 gene scores in the anxiety symptom group, we replicated six genes, including *CNNM2*, *EXD3*, *GBF1*, *NT5C2*, *NOLC1* and *TRIM* (Fig. 3h). Of the six gene scores associated with the emotional instability symptom group, we replicated three genes including *IFT74*, *LDHC* and *TMPO* (Fig. 3k).

### Brain volume differences underlying environmental profiles and psychiatric symptom groups

**Fig. 4:**
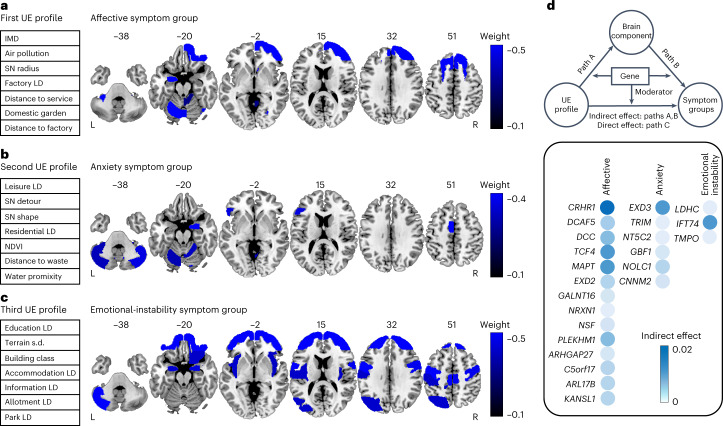
Brain volume differences underlying environmental profiles and psychiatric symptom groups. **a**–**c**, Left, Top urban environmental categories contributing to the first (**a**), second (**b**) and third (**c**) urban living environmental profile in the msCCA regression in the UKB-NI dataset. Right, Regional brain volume maps associated with the three urban living environmental profiles and affective (**a**), anxiety (**b**) and emotional instability (**c**) symptom groups. **d**, Top, Schematic diagram of moderated mediation analysis between genomics, urban environmental profile, brain components and psychiatric symptom groups. Bottom, Each dot shows an indirect effect in the moderated mediation analysis between urban environmental profiles, gene scores, brain components and psychiatric symptom groups. We found that the *CRHR1* (EME = 2.01%), *MAPT* (EME = 1.72%), *TCF4* (EME = 1.71%) and *DCC* (EME = 1.51%) genes moderate the mediation pathway from the first urban environmental profile to brain components of the affective symptom group. The *EXD3* gene moderates the mediation pathway from the second urban environmental profile to brain components of the anxiety symptom group (EME = 1.65%). The *IFT74* gene moderates the mediation pathway from the third urban environmental profile to brain components of emotional instability symptom group (EME = 1.52%).

To investigate differences in brain volume underlying the urban living environment and psychiatric symptoms, we carried out a multiple sparse canonical correlation analysis (msCCA) on the urban living environment profiles, regional brain volume and psychiatric symptom groups. This analysis was conducted in an independent UKB-NI dataset (*n* = 14,988), split into training (90%) and test datasets (10%). We found 13 regional brain volumes significantly associated with the first urban environmental profile (training *r* = −0.050, *P*_perm_ < 0.001; test *r* = −0.042) and the affective symptom group (training *r* = −0.069, *P*_perm_ < 0.001; test *r* = −0.046). Brain volume associations were found in the left amygdala and right ventral striatum, right frontal pole, right occipital fusiform gyrus, as well as bilateral superior frontal cortex, cerebellar lobules VIIIa and VIIb, and right posterior cerebellum Crus I and II (Fig. 4a and Supplementary Table 14). The first urban environmental profile was negatively correlated with brain volume in these areas and positively correlated with the affective symptom group.

We also found 11 regional brain volumes significantly associated with the second urban environmental profile (training *r* = *−*0.015, *P*_perm_ = 0.02; test *r* = *−*0.012) and the anxiety symptom group (training *r* = *−*0.057, *P*_perm_ < 0.001; test *r* = *−*0.045). Brain volumes included the left inferior frontal gyrus, left supplementary motor area and the right amygdala, the bilateral cerebellar lobules VIIIa and VIIIb, bilateral posterior cerebellum Crus I, right cerebellar lobule V and left cerebellar lobule VI (Fig. 4b and Supplementary Table 14).

Finally, 13 regional brain volumes, including the bilateral frontal pole, amygdala, precentral gyrus, insular and left lateral occipital cortex, were associated with the third urban environmental profile (training *r* = *−*0.017, *P*_perm_ = 0.02; test *r* = *−*0.013) and the emotional instability symptom group (training *r* = *−*0.053*, P*_perm_ < 0.001; test *r* = *−*0.040) (Fig. 4c and Supplementary Table 14).

### Moderated mediation of environmental profiles, brain volume and psychiatric symptom groups by genomics

To test whether the relationships between urban living environment profiles with psychiatric symptom groups were mediated by brain volume and moderated by genetic differences, we independently performed moderated mediation analysis for each replicated gene score (moderating variable), three urban living environmental profiles (independent variable), three brain volume components (mediated variable) and three psychiatric symptom groups (dependent variable) in 8,705 adult participants with complete data. Twenty-three moderated mediation analyses were tested (14 gene scores of the affective symptom group, six gene scores of the anxiety symptom group and three gene scores of the emotional instability symptom group). Of the 23 replicated gene scores, the *CRHR1* (explained mediation effect (EME) = 2.01%), *MAPT* (EME = 1.72%), *TCF4* (EME = 1.71%) and *DCC* (EME = 1.51%) gene scores moderate the mediation pathway between the urban environmental profile, brain components and affective symptom group (Fig. 4d and Supplementary Table 15). Specifically, participants with higher *CRHR1* genetic risk living in areas with greater urban environmental exposure had lower brain volume and demonstrated more severe affective symptoms (*β* = 0.02, s.e. = 0.009, 95% lower confidence interval (CI) = 0.006, 95% upper CI = 0.04; Supplementary Table 15). The *EXD3* gene score (EME = 1.65%) moderated the mediation pathway of the anxiety symptom group and the *IFT74* gene score (EME = 1.52%) moderated the mediation pathway of the emotional instability symptom group (Fig. 4d and Supplementary Table 15).

## Discussion

**Fig. 5:**
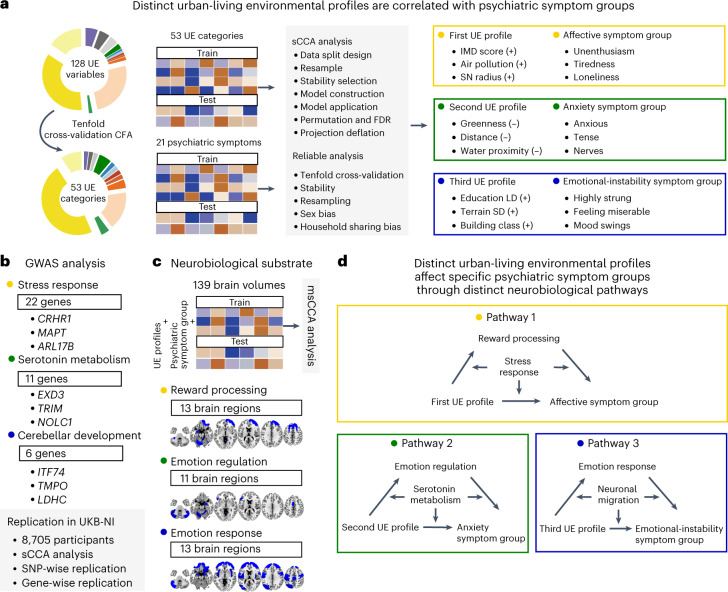
Schematic summary of main findings. **a**, Distinct urban-living environmental profiles are correlated with three psychiatric symptom groups. **b**, GWAS associations and relevent replication analyses reveal that three environmental psychiatric symtom groups are invloved distinct biological pathways. **c**, msCCA analyses revealed that three environmental psychiatric symtom groups were invloved distinct neurobilogical substrate. **d**, Different environmental profiles of urban-living may influence specific psychiatric symptom groups through distinct neurocognitive pathways.

In this study, we describe how urban living affects the brain and mental health by identifying specific environmental profiles that are correlated with distinct groups of affective, anxiety and emotional instability symptoms, mediated by reductions in regional brain volume and moderated by genes involved in pertinent biological pathways (Fig. 5). Whereas previous studies that investigated the relationships between environment, biology and mental health mostly focused on microenvironmental psychosocial factors, we report the discovery of macroenvironmental physical and socioeconomic environment profiles that are linked to psychiatric symptoms. We also developed a unified model capable of integrating multimodal environmental, biological and behavioral components. The model enabled the discovery of complex living environments that affect distinct psychiatric symptom groups and uncovered the underlying biological mechanisms.

Our characterization of multimodal urban environmental profiles that simultaneously enable a qualitative and quantitative assessment of each factor of the profile extends from the isolated assessment of individual environmental factors, as has previously been the norm^26^. Environmental profiles explain a greater degree of variance (4.71%) than comparable studies measuring individual environmental factors, such as nighttime light (2.56%), built-up percentage (1.21%) and NDVI (1.00%)^11^ alone. They enable the assessment of each individual environmental factor in a context that is relevant for mental health. Furthermore, we describe how the effect of urban environmental profiles on psychiatric symptom groups are mediated by regional brain volume and moderated by genetic factors.

By providing evidence for brain-related correlates of environmental adversity, neurobehavioral interventions could be developed to convey adaptive coping skills for environmental adversity, for example, through neurofeedback-guided virtual reality sessions. The identification of genetic moderators suggests differential susceptibility^27^ and identifies biological targets for intervention that might underlie the observed relationships between the urban living environment, brain and psychiatric symptoms.

The first urban environmental profile identified is dominated by high degrees of deprivation and air pollution, and to a lesser extent traffic, short distance to infrastructural facilities and lack of green space. This environmental profile evokes the image of a poor, dense inner-city neighborhood. It is correlated with increased affective symptoms. While there is mixed evidence linking neighborhood socioeconomic conditions with affective disorders^13^, our findings are consistent with a recent meta-analysis reporting the association of poorer socioeconomic conditions with higher odds of depression^28^. The correlation between the first urban environmental profile and the affective symptom group is mediated by volume reductions in brain regions that underlie different functions of reward processing. The ventral striatum is the central area of reward and drug reinforcement^29^. The ability to correctly evaluate different aspects of reward depends on reinforcement learning and emotional recognition involving the cerebellar Crus I and II^30^ and the amygdala^31^, which in turn is influenced by object recognition information from the fusiform gyrus^32^. Activity of the superior frontal gyrus responds to social punishment^33^. The frontal pole and superior frontal cortex switch executive control to new sources of reward^34^. While these findings point toward reward processing as a plausible mediator of a stressful environment on affective symptoms, they are hypothesis-generating only and require further testing. We identified genetic moderators that influence distinct brain and reward mechanisms underlying the affective symptoms identified. *CRHR1*, a critical regulator of the hypothalamic and behavioral extrahypothalamic stress response, which is expressed widely in the human brain, including in the regions forming affective brain correlates^35^. The environmental effect is moderated by genes regulating brain structure, including the predominantly cortical *MAPT*, involved in neurodegeneration^36^, the predominantly cerebellar *TCF4*, inducing neural differentiation^37^, and the mainly subcortical *DCC*, an adhesion molecule that guides axon growth^38^. We also identified moderating genes associated with the affective symptom group involved in relevant neural mechanisms, including G-protein signaling (*ARL17B*)^39^ and epigenetic regulation (*KANSL1*)^40^, both with high cerebellar expression. These genes are located in two genomic loci of chromosome 17q21.3 and 18q21.2. The chromosome 17q21.3 genomic locus is the site of a human supergene candidate of tightly linked functional genetic elements spanning approximately 900 kb that are inherited as a unit^41^. Haplotypes of this cluster are associated with brain morphology and cognitive and depressive behavior, neuroticism and risk-taking behavior^42^. The chromosome 18q21.2 region at the transcription factor 4 gene *TCF4* and netrin 1 receptor gene *DCC* is associated with eight psychiatric disorders^43^, including depression and neuroticism^44,45^. The product of *DCC* guides axonal growth during neurodevelopment and serves as a master regulator of midline crossing and white matter projections^46^. The molecular contribution of these genes to the brain correlates identified requires further investigation.

The second environmental profile captures a different urban living profile that is dominated by green spaces and long distances to waste and energy facilities as well as presence of lakes, rivers and the sea, all of which are inversely correlated (protective) with symptoms of anxiety. The anxiety symptom cluster is also positively correlated with greater density of streets and leisure places as well as urban regions with mixed residential, commercial and industrial use. These correlations point to an important role of green spaces and a more generous land use as protective factors associated with symptoms of anxiety, extending previous findings linking urban green spaces to mental health^11^. The relationship between the second urban environmental profile and anxiety symptoms is mediated by volume reductions in the inferior frontal regions, amygdala and cerebellar regions, including Crus I and lobule VIII. As the amygdala and inferior frontal cortex are part of the prefrontal limbic system, it is possible that the anxiety brain correlate is involved in emotional regulation. The amygdala has a primary role in fear and anxiety responses on activation^47^. The inferior frontal cortex provides evaluation of stimulus meaning to the ventromedial prefrontal cortex, which inhibits amygdala activity^48^. The cerebellar Crus I and lobule VIII are implicated in anxiety vulnerability^49^, possibly through its role in associative learning, modulated by amygdala input^50^. Anxiety brain correlates are moderated by variations in the exonuclease *EXD3* gene, which is involved in nucleic acid binding with the highest expression in frontal cortical areas. *EXD3* is associated with anxiety, phobia and dissociative disorders^51^.

While statistically significant, the third urban living environmental profile, after repeated orthogonalization, explains a decreased amount of variance compared to the previous two profiles, which is common in CCA^19^. Therefore, we do not offer a strong interpretation of the third urban living environmental profile. Its environmental profile shows positive correlations of density of land use and urban infrastructure with a group of emotional instability symptoms, which were mediated by the frontal pole, amygdala, precentral gyrus, insular cortex and cerebellum, and moderated by *ITF74*, a neuronal migration factor associated with schizophrenia^25^.

We found some degree of pleiotropy within psychiatric symptom groups. Urban environmental factors with the greatest degree of pleiotropy explain the smallest amount of variance in all three psychiatric symptom groups. Correspondingly, the stronger a symptom group can be predicted by environmental variables, the less pleiotropic the predictor will be. This specificity might be due to strong environmental predictors being associated with defined biological mechanisms that affect specific behavioral symptoms. Such behavioral symptoms might be observed in different psychiatric disorders, resulting in urban environmental factors conveying their influence in a pervasive and transdiagnostic way. One example in our results is the relation of IMD score (known to increase psychosocial stress^52^) with *CRHR1*-signaling and the affective symptom group. The IMD score was highly predictive of the affective symptom group, with a low degree of pleiotropy within this symptom group, and was not predictive of the anxiety symptom group.

A limitation of our work is potential attrition bias in the different statistical subsets of the UKB cohort^53^. While our linear mediation model suggests a causal effect of urbanicity on behavior that is mediated by the brain, alternative explanations are available. These include selective migration of individuals at high risk of developing psychiatric disorders into socioeconomically deprived urban areas, which may be partly genetically driven^54^, or unmeasured familial factors that account for the association between urbanicity and mental health^55^.

Our data do not characterize the individual biological pathways that mediate defined environmental adversity. To carry out the causal and mechanistic investigations necessary to identify biomarkers for risk and resilience, a deeply phenotyped, longitudinal dataset is required. Our findings generate hypotheses that may be tested in well characterized samples of a much smaller size. Also, the generalizability of our results across ethnicities and beyond industrialized high-income countries requires further investigation.

By providing evidence for comprehensive urban environmental profiles that affect distinct groups of psychiatric symptoms and are mediated by different brain mechanisms, our results characterize biological mechanisms underlying complex, real-life environmental adversity. The quantification of the contribution of each environmental factor to brain and psychiatric symptoms and their interplay in an urban-living environment could potentially aid in targeting and prioritizing future public health interventions.

## Methods

### UKB cohort

The UKB is a population-based cohort including 502,616 participants recruited in the United Kingdom between 2006 and 2010^56^. Participants who were registered with the National Health Service and living within a 40-km radius of one of the 22 assessment centers in England, Wales and Scotland were invited to enter the cohort. Among the 502,616 participants, participants of this study were exclusively adults and aged from 40 to 77; mean age at baseline was 59.46 years (s.d. = 8.12), 54.41% were men and 81.51% were of White ethnicity. The average Townsend Deprivation Index, a measure of regional socioeconomic status, was −1.29 with an s.d. of 3.09, thus showing slightly less deprivation than the UK average (scaled at 0); 11.49–56.51% participants had ever suffered from different psychiatric symptoms at baseline (Supplementary Table 4). The main goals of the UKB are to explore the etiology of common complex diseases by investigating their association with the underlying genetic and lifestyle determinants, which may contribute to the advancement of modern medicine and treatments that improve human health. Baseline assessments included genomics, physical and social exposure, sociodemographics, and lifestyle, occupational, psychosocial and environmental measures. Written informed consent was obtained from all UKB participants.

### Ethics approval

This study was covered by the ethical approval from the UKB granted by the National Information Governance Board for Health and Social Care and the NHS North West Multicenter Research Ethics Committee. All participants provided informed consent through electronic signature at baseline assessment. The data collected at baseline were used in this study. The demographic information of each statistical analysis is shown in Table 1.

### Data collection

#### Urban living environment data

IMD, traffic, air and sound pollution, green space proximity, coastal proximity, water proximity as well as urban morphometric measures were used to measure the urban living social and physical environment around participants available in the category ‘local environment’ in the UKB (data field 113). A total of 53 categories including 128 urban living environment variables were included in the urban living environment data (Extended Data Fig. 3). The detailed variables and categories used are shown in Supplementary Tables 1–3. To exclude variables with extremely skewed data distribution, we used the function nearZeroVar from the caret R package^57^; no variables were excluded. In the 128 variables, we calculated the median absolute deviation (MAD) and removed values larger than 4 MAD in each environment variable. For further analyses, we used 216,341 participants with complete 128 urban living environmental variables.

#### IMD

IMD scores were used to classify the relative deprivation (a measure of poverty) in British local councils published by UK government (https://www.gov.uk/government/collections/english-indices-of-deprivation). IMD scores were calculated separately in England (EIMD), Scotland (SIMD) and Wales (WIMD) because multiple different components of deprivation are weighted with different strengths and compiled into a single score of deprivation. The EIMD score consists of seven domain indices, including: income deprivation (income subdomain, income deprivation affecting children index and older people index); employment deprivation; health deprivation and disability; education score; barriers to housing and services (wider and geographical barriers subdomain); living environment deprivation (indoors and outdoors subdomain); and crime score. The SIMD score consisted of seven domain indices, including: crime (only from 2006), current income, education, skills and training, employment, geographical access, health and housing. The WIMD score is composed of eight domain indices for income, employment, health, education, access to services, community safety, physical environment and housing.

#### Traffic

Traffic consists of seven items: (1) close to major road; (2) inverse distance to the nearest major road; (3) inverse distance to the nearest road; (4) sum of road length of major roads within 100 m; (5) total traffic load on major roads; (6) traffic intensity on the nearest major road; (7) traffic intensity on the nearest road.

#### Air pollution

Residential air pollution consists of six items: (1) nitrogen dioxide air pollution from 2005 to 2010; (2) nitrogen oxide air pollution in 2010; (3) particulate matter 10 μm air pollution in 2007 and 2010; (4) particulate matter 2.5 μm air pollution in 2010; (5) particulate matter 2.5 μm air pollution absorbance in 2010; and (6) particulate matter 2.5–10 μm air pollution in 2010.

#### Sound pollution

Residential sound pollution consists of five items: (1) average 16-h sound level of noise pollution; (2) average 24-h sound level of noise pollution; (3) average daytime sound level of noise pollution; (4) average evening sound level of noise pollution; (5) average nighttime sound level of noise pollution.

#### Green space proximity

The green space proximity category contains environmental indicators relating to green space exposure attributed to participants based on 300-m home location buffers, including three items: (1) natural environment percentage estimate compared to the ‘built environment’; (2) green space percentage estimates; (3) domestic garden percentage estimates.

#### Coastal proximity

The coastal proximity category contains environmental indicators of distance from home location to the coast, which was attributed to participants based on 300-m home location buffers.

#### Water proximity

The water proximity category contains environmental indicators of domestic water percentage estimates, which was attributed to participants based on 300-m home location buffers.

#### UKB Urban Morphometric Platform measures

The UKB Urban Morphometric Platform (UKBUMP) is a high-resolution spatial database of urban morphological metrics within residential street catchments of the geocoded home address of UKB participants^58^. UKBUMP aims to provide a national platform for evidence-based healthy city planning and public health interventions. The platform will facilitate the construction of models that will explicitly decipher health impacts, from genetic to micro-built environment scales. Specifically, spatial and network modeling were performed on multiple UK-wide datasets, including the AddressBase Premium data of the Ordnance Survey GB, remotely sensing data, digital terrain topographical models and other datasets based on the anonymized geocoded home address of UKB participants. A total of six metrics including 104 urban living environment variables from the UKBUMP dataset were used. The six metrics used in this study include: (1) building class (*n* = 1): building class was extracted for the area of interest and building footprints were subsequently linked with the geocoded residences of UKB participants through a spatial query, so that each UKB participant’s dwelling fell within one of the six building age code categories and 19 building type code categories. The age and type codes were combined together to form the building class code of each dwelling, which we used here; (2) destination accessibility (*n* = 33): health-specific destination accessibility was derived as a part of the morphometric analysis of the built environment, which was measured in the form of network distance from a respondent’s dwelling to the nearest 33 different destinations (such as GP practice, dentist, library, hospital, post office); (3) NDVI (*n* = 2): greenness was measured by an objective measure, namely the NDVI. The NDVI is a unitless index calculated from the reflectance measures in color infrared remote sensing satellite data, comparing the amount of energy absorbed by the chlorophyll in the red portion and the amount scattered by the internal structure of the leaves in the near-infrared region. The index ranges from −1 to +1, with higher values reflective of healthy green vegetation and vice versa. Greenness was calculated in terms of mean, minimum, maximum and s.d. in the NDVI values within the defined 500 m and 1,000 m around the address of residence of each UKB participant. We used the mean and s.d. of NDVI based on 500-m home location buffers; (4) LD (*n* = 46): LD was measured for different land use classes within 500, 1,000, 1,500, 2,000-m SN catchments of each UKB respondent’s dwelling and within the lower super output areas in which they resided. We used the 46 LD categories based on 500-m home location buffers; (5) SN (*n* = 20): the physical accessibility of SN was modeled through spatial design network analysis (sDNA). sDNA is a sophisticated technique of urban network analysis that has evolved from conventional network analyses and uses SN links as the fundamental unit of computation^59^. The Ordnance Survey MasterMap Integrated Transport Network was subjected to automated cleaning in the sDNA Prepare Tool; subsequent modeling produced a suite of 18 different indices of SN accessibility (Supplementary Table 3). These measure the link, centrality, radial, detour and shape characteristics of urban morphology captured at the micro (neighborhood), meso (city) and macro (regional) level encompassing 19 different catchment radii (400–50,000 m). In this study, we only used the measures with a catchment radius of 400 m (Supplementary Table 3). These are generated in sDNA for all links in the urban road network covering the entire UKB cohort; the metrics for a street link containing a UKB respondent’s dwelling were added to the respondent’s built environment profile. The detailed description of these SN morphometric measures is shown in Supplementary Table 3; and (6) terrain (slope) (*n* = 2): slope analysis was conducted in Spatial Analyst, ArcGIS v.10.2 using a digital terrain model. Terrain was calculated in terms of mean, minimum, maximum and s.d. in the terrain slope values within the defined 500 m and 1,000 m] around the address of residence of each each UKB participant. We used the mean and s.d. of terrain value based on 500-m home location buffers.

#### Psychiatric symptoms

There are 44 psychiatric symptoms in the category ‘mental health’ in the UKB that cover symptoms of affective and anxiety disorders, as well as personality (category ID: 100060). These items were obtained from a standardized mental health questionnaire that participants answered at the time of recruitment. Of this questionnaire, 21 items were excluded because the missing rate was greater than 50% from 502,616 participants of the UKB. To exclude psychiatric symptoms with extremely skewed data distribution, we used the function nearZeroVar from the caret R package and excluded two items. Finally, a total of 21 psychiatric symptoms with complete data in 365,201 participants were included in the further analysis. A full list of the 21 psychiatric symptoms is shown in Supplementary Table 4.

#### Genomics data

We used the imputed genomic data (v.3) made available by the UKB for 487,411 individuals^60^, which was imputed from the Haplotype Reference Consortium reference panel^61^ and a merged UK10K and 1000 Genomes phase 3 reference panel^62^. Using participant-level quality control, we applied exclusion filters for participants as follows: (1) participants with a mismatch in reported sex and chromosome X imputed sex or with putative sex chromosome aneuploidy; (2) participants with genetic kinship to other participants; (3) participants with excess heterozygosity or missing rates; (4) non-White participants; (5) participants without calculated genetic principal components. Using SNP-level quality control, we applied exclusion filters for SNPs as follows: (1) minor allele frequency < 0.001; (2) imputation INFO quality score > 0.3. A total of 275,988 participants and 13,918,727 SNPs were used in the further analysis.

#### Neuroimaging data

In this study, neuroimaging data were acquired from one 3 Tesla magnetic resonance imaging scanner from Siemens (Skyra running VD13A SP4 with a standard 32-channel radiofrequency receive head coil) at the UKB imaging center in Manchester. The standard parameters of a 3D MPRAGE sequence can be accessed at https://biobank.ctsu.ox.ac.uk/crystal/crystal/docs/brain_mri.pdf. FAST gray matter segmentation was used to generate a further 139 regional image-derived phenotypes by summing the gray matter partial volume estimates within 139 regions of interest (ROIs): 111 cortical and subcortical gray matter volume (GMV) and 28 cerebellum GMV (field ID: 1101). These ROIs are defined in the MNI152 space, combining parcellations from several atlases: the Harvard-Oxford cortical and subcortical atlases (https://fsl.fmrib.ox.ac.uk/fsl/fslwiki/Atlases) and the Diedrichsen cerebellar atlas (http://www.diedrichsenlab.org/imaging/propatlas.htm). The detailed information can be accessed at https://biobank.ctsu.ox.ac.uk/crystal/crystal/docs/brain_mri.pdf. The neuroimaging data for a total of 42,796 participants were used for the present study.

#### Confounding variables

Age, sex and assessment centers were adjusted as confounding covariates in the further analysis. A total of 502,616 participants had complete confounding variables. The 21 psychiatric symptoms were first corrected for confounding variables and then normalized. For the neuroimaging-related analyses, total intracranial volume was also corrected.

### Statistical analysis

#### Train and test sample split design

Participants from the UKB with complete urban living environmental data and psychiatric symptoms (*n* = 156,075) were divided into UKB-non-NI (*n* = 141,087) and UKB-NI (*n* = 14,988) datasets. The UKB-non-NI dataset was divided into training and test datasets to ensure validity of the results: 90% of participants were used as a training dataset (*n* = 126,978) and 10% of participants (*n* = 14,109) were used as a test dataset for model validation. The UKB-NI dataset (*n* = 14,988) was used for independent replication of the relationship between urban living environment, genomics and psychiatric symptoms, and for additional neuroimaging analyses (Table 1, Fig. 1 and Extended Data Fig. 1).

#### Construction of urban living environmental categories

We included 53 urban living environmental categories consisting of 128 variables in the study (Fig. 2a). Among these, 34 categories had one independent environmental item. In the remaining categories, redundancy between related environmental items was avoided by collapsing the information into 19 latent environmental categories using tenfold cross-validation CFA using the lavaan R package (https://cran.r-project.org/web/packages/lavaan) (Extended Data Fig. 3). In the CFA models, tenfold cross-validation was performed to ensure unbiased estimates of generalizability throughout the analytical pipeline and to optimize the CFA models. For each fold, 90% of participants were used to build the CFA model; the optimized CFA model was then used to calculate latent variables for the remaining 10% of participants in each environmental subcategory. We used two criteria to optimize the CFA model by selecting appropriate environmental measures. The first criterion was the goodness of fit of the CFA model assessed by Tucker–Lewis index (TSI), comparative fit index (CFI), chi-squared, root mean square error of approximation (RMSEA) and standard root mean square residual (SRMR). Criteria for an excellent model fit were TSI > 0.95, CFI > 0.95, RMSEA < 0.06 and SRMR < 0.08. The second criterion was the inclusion of environmental measures that best reflected different aspects of the urban environment. For example, in residential sound pollution variables, we initially constructed a CFA model by including all five sound pollution measures in the training dataset. Based on the factor loadings of the five sound pollution measures, we removed the ‘average nighttime sound level of noise pollution’ item with the smallest factor loading and repeated the CFA modeling. These steps were iterated until the resulting CFA model satisfied our criteria for excellent model fit in the training dataset. The factor loadings of the optimized CFA model were used to calculate the latent residential sound pollution measure in the test dataset. This process was applied into ten folds to predict all out-of-sample 19 urban living environmental categories.

#### Multivariate relation of urban living environmental profiles with psychiatric symptoms

To investigate the multivariate relationship between urban living environment and psychiatric symptoms, we conducted multivariate analyses using sCCA with the sgcca.wrapper function of the mixOmics R package based on our previous work^63^. The analysis design was carried out as follows: (1) the full dataset was randomly split into training and test datasets. The training dataset consisted of 90% of the data while the testing set consisted of the remaining 10%; (2) the training dataset was then randomly split into 100 resamples. Each resample consisted of *n*_*t*_/2 participant scans, where *n*_*t*_ is the total number of participants in the training dataset; (3) the first stage of the msCCA regression algorithm was then applied to each resample, with a sparsity constraint of 0.5 in each view of the data^17^; (4) the resulting weights for each urban environmental category and psychiatric symptom(s) were recorded for each resample. The urban environmental category and psychiatric symptom(s) with non-zero loading greater than 90% across the resamples were selected and retained as stable variables in subsequent analyses. Stability selection was also applied to limit false discoveries by selecting only variables that were stable under resampling. This resulted in a reduction in the number of variables necessary to achieve equivalent predictive performance and properly accounts for correlations between them. Consequently, stability selection effectively prevents variable categories with many candidate predictors from ‘overwhelming’ categories with fewer candidate predictors; (5) we then reapplied the sCCA algorithm to the data, without sparsity constraints, on the stable urban living environment category and psychiatric symptom(s) in the training dataset. The canonical correlation *r* value between urban living environment category and psychiatric symptom(s) were recorded; (6) we then permuted the training data and repeated steps 2–5. This was done for 1,000 different permutations of the training data labeling. In each case, we recorded the canonical correlation *r* value between urban living environment category and psychiatric symptom(s). Thus, we built up a permutation distribution to assess the significance of the relationship between urban living environment category and psychiatric symptom(s) in the experimental labeling within the training dataset; (7) we then applied the trained model to the test dataset to produce canonical correlates of urban living environment (which we refer to as ‘urban living environment profile’) and psychiatric symptom(s) (referred to as ‘psychiatric symptom groups’). We recorded the canonical correlation *r* value for the training and testing datasets; and (8) we then randomly permuted the data rows in the test dataset and recalculated the *r* values between urban living environment profile and psychiatric symptom groups for each of 1,000 permutations of the experimental labeling. The *P*_perm_ demonstrated the *P* value for a one-tailed permutation test (*P*_perm_) in the training and test datasets. False discovery rate (FDR) correction was used to control for multiple testing and a *P*_FDR_ = 0.05 was considered statistically significant.

#### Finding multiple modes between urban living environmental profiles and psychiatric symptom groups

After determining the significance of the first canonical correlate, we removed the effect of the first set of canonical vectors using projection deflation^17,18,24^. This is important to keep in mind to correctly interpret our findings, in particular the GWAS analysis. We then repeated the analysis to investigate the presence of a second canonical correlation that explains covariance over and above what is explained by the first component. These steps were iterated until the resulting canonical correlates were no longer statistically significant. Finally, we calculated the canonical correlation *r* value between the urban living environment profile and psychiatric symptom groups, the weight value of each environmental category and psychiatric symptom variables, and the explained variance and fraction of explained variance of crossloading of each urban living environmental category for each psychiatric symptom group in each mode.

#### Reliability analyses

We undertook the following analyses to evaluate the robustness and reliability of the results in the sCCA analysis between urban living environment profiles and psychiatric symptoms: (1) sCCA stability: to assess the stability of sCCA in relation to sample size and composition, we performed a sensitivity analysis using bootstrapping by rerunning the algorithm in 100 randomly generated subsamples, each containing 10–150% of the training dataset in 10% increments with replacement, recalculating the canonical correlation between urban living environmental profiles and psychiatric symptoms; (2) random resampling: we performed a sensitivity analysis using bootstrapping to resample the training data with replacement 1,000 times, each containing 10–150% of the training dataset in 10% increments. We resampled 90% of the training dataset 1,000 times, reran the sCCA algorithm and calculated the canonical correlation between the resulting feature loadings in the remaining 10% of the training dataset each time; (3) Sex bias: to evaluate whether there was similarity of the original sCCA modes between males and females, we calculated the canonical correlation between urban living environment and psychiatric symptoms in males and females separately. The canonical correlation of males and females was then calculated for all three significant modes in both training and test datasets; and (4) Household sharing bias: the urban environment variable was measured at the individual level and connected to each participant’s address. However, there were genetically unrelated participants living in the same household. To rule out the influence of genetically unrelated members of the same household, we reperformed the sCCA regression to exclude the household sharing bias. Household sharing was not explicitly available; therefore, we used similar methods to previous studies^64^ to identify potential household sharing participants in the UKB. The household sharing information was used to extract participants who (1) reported living with their spouse (field ID: 6141); (2) reported the same length of time living in the house (field ID: 699); (3) reported the same number of occupants in the household (field ID: 709); (4) reported the same number of vehicles (field ID: 728); (5) reported the same accommodation type and rental status (field IDs: 670 and 680); (6) had identical home coordinates (rounded to the nearest km) (field IDs: 20074 and 20075); and (7) were registered with the same UKB recruitment center (field ID: 54). If more than two participants shared identical information across all variables, these participants were potentially regarded as being in the same household. We identified 36,071 potential household sharing participants belonging to 17,500 independent households in 141,087 participants with complete urban environmental measures and psychiatric symptom groups in the UKB-non-NI dataset. We randomly kept one participant from the potentially same household. Finally, 18,571 participants were excluded and 122,516 participants were used to reperform the sCCA between urban environmental variables and psychiatric symptom groups.

#### Genome-wide-significant associations with environmental psychiatric symptom groups

For the significant psychiatric symptom groups that correlated with urban living environment profile from the sCCA results, we conducted a GWAS of the corresponding psychiatric symptom groups in 76,508 participants with complete genomic, urban environment and psychiatric symptoms data (Table 1). Using BGENIE v.1.2 (https://jmarchini.org/bgenie/), we fitted an additive model of association at each variant, using the expected genotype count (dosage) from the imputed genetic data. The covariates included age, sex, assessment center, processing batch and the top 10 ancestry principal components. Bonferroni-corrected *P* < 0.05 (uncorrected *P* < 0.05/13,918,727 × numbers of significant psychiatric symptom groups from the sCCA results) was considered as a statistically significant threshold. All SNPs with genome-wide significance were mapped to genes based on physical distance from the human reference assembly (GRCh37/hg19) using the FUMA portal (https://fuma.ctglab.nl/).

#### GSEA

To better understand the biological function of fine-mapped genes associated with psychiatric symptom groups, these genes were functionally annotated using the ToppGene portal (https://toppgene.cchmc.org/) to identify significant enrichments for gene ontology (GO). Bonferroni correction (*Q* < 0.05) was applied to correct for multiple comparisons. The default full reference gene list of each category in ToppGene was used as the background gene set. The Human Protein Atlas portal (https://www.proteinatlas.org/) was additionally used to identify gene overexpression in 12 brain regions for moderating genes.

#### Gene score calculation

We created a score for the genes associated with each psychiatric symptom group, using PLINK 2.0 (ref. ^65^) with default parameter settings adjusted for linkage disequilibrium. Specifically, the clump-p1-indicated GWAS *P* threshold for an SNP to be included as an index SNP was set to 1 such that all SNPs were include for clumping. Clump-r2 was set to 0.5, indicating that SNPs having an *r*^2^ greater than 0.5 with the index SNPs would be removed. Clump-kb was set to 250 kb, indicating that SNPs within 250k of the index SNP were considered for clumping. The score of each gene was then calculated as the sum of the count of risk alleles multiplied by the corresponding *β* value from the GWAS across the remaining index SNPs. Thus, we generated 22 gene scores associated with the affective symptom group, 11 gene scores for the anxiety symptom group and six gene scores for the emotional instability symptom group.

#### Replication in the UKB-NI dataset

To replicate the multivariate relationship between urban living environment and psychiatric symptoms, we applied the sCCA analysis in an independent dataset of 14,988 participants with complete environmental, mental health and neuroimaging data from the UKB-NI dataset. Again, we used a training dataset (*n* = 13,490, 90%) and a test dataset (*n* = 1,498, 10%), a resampling method to ensure variable stability (with a threshold of 90% for non-zero weights from resampled data to consider as stable variables) and permutation tests to assess the significance of the results (10,000 times) as used in the discovery sCCA analysis. Next, we independently replicated the significant SNPs associated with psychiatric symptom groups surviving from the discovery GWAS analysis (UKB-non-NI dataset) in an independent 8,705 participants of the UKB-NI dataset at a Bonferroni-corrected *P* < 0.05 (uncorrected *P* < 0.05/the numbers of all significant SNPs of the GWAS of psychiatric symptom groups from the discovery analysis). Then, we calculated the corresponding gene scores as we did in the discovery analysis. Finally, we independently validated the associations between gene scores and psychiatric symptom groups in the UKB-NI dataset.

#### Brain volume differences are correlated with urban living environmental profiles and psychiatric symptom groups

To investigate the neurobiological mechanisms underlying the associations between urban living environment and mental health, we carried out an msCCA between the urban environmental profiles, brain volume measures and psychiatric symptom groups using the mixOmics R package. This analysis was conducted in an independent sample of 14,988 participants with urban environmental, mental health and neuroimaging data from the UKB-NI dataset. Again, we used a training dataset (*n* = 13,490, 90%) and a test dataset (*n* = 1,498, 10%), a resampling method to ensure variable stability (with a threshold of 85% for non-zero weights from resampled data to consider as stable variables) and permutation tests to assess the significance of the results (10,000 times). Finally, the brain volume canonical variables (referred to as brain component), the canonical correlation *r* value between urban environmental profile and brain component, the canonical correlation *r* value between brain component and psychiatric symptom groups, and the weights of the corresponding regional brain volume variables were calculated.

#### Moderated mediation analysis between urban living environmental profile, brain components and psychiatric symptom groups modulated by genomics

The above analysis was carried out separately to identify the associations of urban living environment with psychiatric symptoms, genetic variation and brain volume, leaving the complex associations of urban living environment, genomics, brain component and psychiatric symptoms unexplored. To formally test whether the urban living environment and psychiatric symptom relationship can be mediated by brain components and modulated by genomics, we carried out a modulated mediation analysis in 8,705 participants using Model 59 in the process R package^66^. Moderated mediation analysis is an extension of mediation analysis, a valuable technique for assessing whether an indirect effect is conditional on a moderating variable. The bases of moderation and mediation effect were integrated into a combined model of moderated mediation within a linear regression framework. Finally, gene scores were defined as modulated variables, the urban living environment profile was defined as an independent variable, the brain component was defined as a mediator variable and the psychiatric symptom groups were defined as a dependent variable. The modulator (gene score) was defined by the 16th, 50th and 84th percentiles as the low, medium and high genetic risk based on the default parameter in the process R package^66^.

In the moderated mediation analyses, all indirect effects were estimated in one multiple regression analysis with independent variables as predictor variables. We used a nonparametric bootstrapping method to assess the significance of the mediation effect. After 5,000 bias-corrected bootstrapping, we estimated the distribution of the indirect effect and calculated its 95% CI. If zero did not fall between the resulting 95% CI of the bootstrapping method, we confirmed the existence of a significant mediation effect (*P* < 0.05). In the multiple mediation analysis of this study, mediators and dependent variables were measured contemporaneously, thus not allowing the establishment of any causal directionality. The EME and 95% CI were reported for the moderated mediation analyses. Confounding factors were controlled in the moderated mediation model.

### Reporting summary

Further information on research design is available in the Nature Portfolio Reporting Summary linked to this article.

## Online content

Any methods, additional references, Nature Portfolio reporting summaries, source data, extended data, supplementary information, acknowledgements, peer review information; details of author contributions and competing interests; and statements of data and code availability are available at 10.1038/s41591-023-02365-w.

## Supplementary information

Supplementary Information Supplementary Tables 1, 3, 4, 6–8 and 14 and members list of the environMENTAL Consortium.

Reporting Summary

Supplementary Tables Supplementary Tables 2, 5, 9–13 and 15.

## Data Availability

All the UKB data used in the study are available at the UKB (https://www.ukbiobank.ac.uk). The Human Protein Atlas portal can be accessed at https://www.proteinatlas.org/.

## Code availability

The core code used to run the analyses reported in this study can be found at https://github.com/jiayuanqqxu/scca-regression.
