# Supplementary material for: Effects of urban living environments on mental health in adults
Source: Nat Med. 2023 Jun 15;29(6):1456–67. doi: 10.1038/s41591-023-02365-w (PMC10287556; doi:10.1038/s41591-023-02365-w)
Supplement: Supplementary file 1 — Supplementary Tables 1, 3, 4, 6–8 and 14 and members list of the environMENTAL Consortium. [file 41591_2023_2365_MOESM1_ESM.pdf]

nature medicine

Article https://doi.org/10.1038/s41591-023-02365-w

Effects of urban living environments on mental health in adults

In the format provided by the authors and unedited

Supplementary Information

Contents

Supplementary Table 1. Urban living environmental variables and categories. 2

Supplementary Table 2. Detailed urban living environmental variables. (see Excel) 4

Supplementary Table 3. Detailed description of street accessibility indices 5

Supplementary Table 4. Details of 21 psychiatric symptoms in UK biobank 8

Supplementary Table 5. Weight of urban living environment category and psychiatric symptoms in sCCA-regression analysis (see Excel) 13

Supplementary Table 6. Multiple comparisons of canonical correlation *r* value in each resampling of training dataset. 14

Supplementary Table 7. Statistics in ten folds cross-validation in sCCA analysis. 16

Supplementary Table 8. sCCA analysis between urban living environment and psychiatric symptoms in males and females, separately 17

Supplementary Table 9. 3,436 significant GWAS associations between SNPs and affective symptoms group after Bonferroni correction of *Pc*<0.05 (see Excel) 18

Supplementary Table 10. 29 significant GWAS associations between SNPs and anxiety symptoms group after Bonferroni correction of *Pc*<0.05 (see Excel) 18

Supplementary Table 11. 10 significant GWAS associations between SNPs and emotional instability symptoms group after Bonferroni correction of *Pc*<0.05 (see Excel) 18

Supplementary Table 12. Gene-set enrichment analysis in gene ontology of affective, anxiety and emotional instability symptom groups associated genes after Bonferroni correction of *Pc*<0.05 (see Excel) 18

Supplementary Table 13. Replications of gene scores associated with psychiatric symptom groups in UKB-NI dataset (see Excel) 18

Supplementary Table 14. Weights for brain volumes simultaneously associated with urban living environment profiles and psychiatric symptoms groups in msCCA-regression. 19

Supplementary Table 15. Moderated mediation statistics in genes scores, urban environmental profile, brain component and psychiatric symptoms groups after bootstrapping (see Excel) . 20

Members list of the environMENTAL Consortium 21

Supplementary Tables

Supplementary Table 1. Urban living environmental variables and categories.

Area	Category	No. of subcategory	No. of variable	Sample size (n)
Traffic	Traffic	1	7	461,291
Air pollution	Air pollution	1	6	461,291
Sound pollution	Sound pollution	1	5	461,291
Greenspace proximity	Natural environment	1	1	497,519
	Greenspace	1	1	440,851
	Domestic garden	1	1	440,851
Water proximity	Domestic water	1	1	440,851
Coastal proximity	Distance from home to coast	1	1	497,519
IMD*	EIMD	1	1	424,419
	SIMD	1	1	424,419
	WIMD	1	1	424,419
Building Class	Building class	1	3	317,095
Terrain	Terrain	2	2	430,832
Destination accessibility	Destination accessibility	9	33	423,886

NDVI	NDVI	2	2	430,832
Landuse density	Landuse density	25	46	424,028
Street network	Street network	5	18	423,562
Total	15	53	128	216,341

EIMD, England Indices of multiple deprivation; SIMD, Scotland Indices of multiple deprivation; WIMD, Wales Indices of multiple deprivation; NDVI, normalized difference vegetation index.

** Each participant only has one IMD score based on their residential locations in England, Scotland and Wales.*

Supplementary Table 2. Detailed urban living environmental variables. (see Excel)

Supplementary Table 3. Detailed description of street accessibility indices

Variable name	Description
**Link characteristics: These measures describe the characteristics of individual links in the network**	
1. Link Connectivity	The number of link ends that an individual link is connected to at its end points
2. Link Length	Length of the individual link in the network
3. Link Angular Curvature	The cumulative angular change while traversing the full length of a link in degrees
**Centrality analysis: These set of measures owe their origin to the graph theory. The associations between urban morphology and the social phenomena dependent on it are essentially captured by indices of relationality in the graphs. The notion of accessibility captured by these measures acts to formally elucidate how network morphology influences individual activity behaviours and drives various socio- economic processes. They indicate the centrality of a vertex within a graph**	
1. Mean Angular Distance	In graphical terminology, also called as the closeness centrality/accessibility. It is an indicator of the degree of difficulty, on average, of navigating to all possible destinations within a specified radius from each given link. This is weighted by the link length
2. Network Quantity Penalized for Distance	This is an improved measure of the conventional closeness centrality and takes in to account the effects of network quantity. For each link within a specified radius, it takes the network quantity (defined link length) and divides it by the difficulty of access (angular). This is weighted by the link length.
3. Betweenness	In graphical terminology, also called as the betweenness centrality or path overlap or through-movement potential. It is indicative of how often a given link is used for a journey within a defined radius. Measured as the sum of geodesics that pass through a link for a journey within a defined radius. This has been weighted by origin-destination link length.

4.	Two Phase Betweenness	This is betweenness weighted by a two-step floating catchment model. Measured as the sum of geodesics that pass through a link for a journey within a defined radius weighted by the proportion of network quantity accessible from geodesic origin that is represented by geodesic destination
5.	Two Phase Destination assignment	This is the total flow to each destination under the two phase betweenness model. In other words, it is similar to the two phase betweenness, but measured for the destination of each geodesic only

Simple radial measures: These measures pertain to the characteristics of the links within a specified network radius.

1.	Links	The number of network links within a specified network radius
2.	Length	The total network length within a specified network radius
3.	Angular Distance	Sum of angular distance of each individual link within a specified radius
4.	Weight	Total weight within a specified radius. Weights have been specified with respect unit of network length (in length weighted analysis)
5.	Mean Geometric Length	Mean of the angular geodesic Euclidean length within a specified radius. This has been weighted by the origin to destination link length

Network detour analysis: Measure the network severance by comparing the hypothetical crow fly distance to actual network distance. It is an indicator of the extent of deviation of the network from the most direct path.

1.	Mean Crow Flight Distance	Mean of the crow flight distance between a link and all the links within a defined radius. This is weighted by the link length
2.	Diversion Ratio	Mean of the ratio of actual geodesic length to the crow flight distance for all geodesics within a defined radius. This is weighted by the link length. Indicative of the degree of deviation of the actual paths from the crow flight path

Network shape: Measure of network efficiency in terms of the spatial footprint of the street network in urban space.

1.	Convex Hull Area	Area of the convex hull containing all the origins and destinations within a defined radius. It is an indicator of the network footprint or the spatial spread of the street network in the urban space.
2.	Convex Hull Perimeter	Length of perimeter of the convex hull containing all the origins and destinations within a defined radius.<br>400,
3.	Convex Hull Maximum Radius	Maximum radius of the convex hull measured as the crow flight distance from the centre of the origin link to the furthest point on the convex hull of a defined radius
4.	Convex Hull Bearing	Compass bearing of the line of maximum radius of convex hull of a defined radius, measured in degrees. It indicates the direction in which one can travel furthest from the origin link, while staying inside the network radius.
5.	Convex Hull Shape Index	Measures the degree of uniformity of the network in all directions. It is measured as the square of the hull perimeter divided by 4π times the hull area. Ranges from 1 in case of a circle to higher values, with higher indicating non-uniformity across all directions.

Supplementary Table 4. Details of 21 psychiatric symptoms in UK biobank

Field ID	Variable name	Question	Answer categories	Sample size	Incidence
1920	Mood swings	Does your mood often go up and down?	1. Yes<br>2. No<br>3. Do not know<br>4. Prefer not to answer	488,298	45.58%
1930	Miserableness	Do you ever feel 'just miserable' for no reason?	1. Yes<br>2. No<br>3. Do not know<br>4. Prefer not to answer	492,219	42.80%
1940	Irritability	Are you an irritable person?	1. Yes<br>2. No<br>3. Do not know<br>4. Prefer not to answer	478,082	28.01%
1950	Sensitivity hurt feelings	Are your feelings easily hurt?	1. Yes<br>2. No<br>3. Do not know<br>4. Prefer not to answer	486,281	55.47%
1960	Fed up feelings	Do you often feel 'fed-up'?	1. Yes<br>2. No<br>3. Do not know<br>4. Prefer not to answer	490,044	40.64%
1970	Nervous feelings	Would you call yourself a nervous person?	1. Yes<br>2. No<br>3. Do not know	487,683	23.63%

			4. Prefer not to answer		
1980	Anxious feelings	Are you a worrier?	1. Yes<br>2. No<br>3. Do not know<br>4. Prefer not to answer	487,727	56.51%
1990	Tense /highly strung	Would you call yourself tense or 'highly strung'?	1. Yes<br>2. No<br>3. Do not know<br>4. Prefer not to answer	482,778	17.76%
2000	Worry too long	Do you worry too long after an embarrassing experience?	1. Yes<br>2. No<br>3. Do not know<br>4. Prefer not to answer	480,269	47.71%
2010	Suffering from nerves	Do you suffer from 'nerves'?	1. Yes<br>2. No<br>3. Do not know<br>4. Prefer not to answer	481,980	21.15%
2020	Loneliness and isolation	Do you often feel lonely?	1. Yes<br>2. No<br>3. Do not know<br>4. Prefer not to answer	492,739	18.56%
2030	Guilty feelings	Are you often troubled by feelings of guilt?	1. Yes<br>2. No<br>3. Do not know<br>4. Prefer not to answer	487,212	28.86%

2040	Risk taking	Would you describe yourself as someone who takes risks?	1. Yes<br>2. No<br>3. Do not know<br>4. Prefer not to answer	482,170	26.94%
2050	Depressed mood	Over the past two weeks, how often have you felt down, depressed or hopeless?	1. Not at all<br>2. Several days<br>3. More than half the days<br>4. Nearly every day<br>5. Do not know<br>6. Prefer not to answer	478,435	-
2060	Unenthusiasm	Over the past two weeks, how often have you had little interest or pleasure in doing things?	1. Not at all<br>2. Several days<br>3. More than half the days<br>4. Nearly every day<br>5. Do not know<br>6. Prefer not to answer	482,800	-
2070	Tenseness	Over the past two weeks, how often have you felt tense, fidgety or restless?	1. Not at all<br>2. Several days<br>3. More than half the days<br>4. Nearly every day<br>5. Do not know<br>6. Prefer not to answer	480,413	-
2080	Tiredness	Over the past two weeks, how often have you felt tired or had little energy?	1. Not at all<br>2. Several days	485,357	-

			3. More than half the days<br>4. Nearly every day<br>5. Do not know<br>6. Prefer not to answer		
2090	Seen a doctor (GP)	Have you ever seen a general practitioner (GP) for nerves, anxiety, tension or depression?	1. Yes<br>2. No<br>3. Do not know<br>4. Prefer not to answer	501,704	33.78%
2100	Seen a psychiatrist	Have you ever seen a psychiatrist for nerves, anxiety, tension or depression?	1. Yes<br>2. No<br>3. Do not know<br>4. Prefer not to answer	501,704	11.49%
6145	Grief and stress	In the last 2 years have you experienced any of the following?	1. Serious illness, injury or assault to yourself<br>2. Serious illness, injury or assault of a close relative<br>3. Death of a close relative<br>4. Death of a spouse or partner<br>5. Marital separation/divorce<br>6. Financial difficulties<br>7. None of the above<br>8. Prefer not to answer	497,926	-

20127	Neuroticism score	The score was generated by summarizing the number of Yes answers across these twelve neurotic behaviour domains into a single integer score for each participant (0 to 12):<br>1. Does your mood often go up and down?<br>2. Do you ever feel 'just miserable' for no reason?<br>3. Are you an irritable person?<br>4. Are your feelings easily hurt?<br>5. Do you often feel 'fed-up'?<br>6. Would you call yourself a nervous person?<br>7. Are you a worrier?<br>8. Would you call yourself tense or 'highly strung'?<br>9. Do you worry too long after an embarrassing experience?<br>10. Do you suffer from 'nerves'?<br>11. Do you often feel lonely?<br>12. Are you often troubled by feelings of guilt?	For each question, participants could answer:<br>1. Yes<br>2. No<br>3. Do not know<br>4. Prefer not to answer	401,652	-

Supplementary Table 5. Weight of urban living environment category and psychiatric symptoms in sCCA-regression analysis (see Excel)

Supplementary Table 6. Multiple comparisons of canonical correlation *r* value in each resampling of training dataset.

**Category**	**Mean**	**Mean difference**	**95% CI**	**95% CI**	$P_{two\text{-}sided}$
100% vs. 10%	0.2156	-0.009357	-0.01088	-0.007837	***<0.0001***
100% vs. 20%	0.2120	-0.005734	-0.007255	-0.004214	***<0.0001***
100% vs. 30%	0.2094	-0.003153	-0.004673	-0.001632	***<0.0001***
100% vs. 40%	0.2082	-0.001956	-0.003477	-0.0004359	***0.0037***
100% vs. 50%	0.2074	-0.001175	-0.002695	0.0003456	0.2320
100% vs. 60%	0.2071	-0.0008983	-0.002419	0.0006221	0.5591
100% vs. 70%	0.2072	-0.0009570	-0.002477	0.0005635	0.4768
100% vs. 80%	0.2070	-0.0007446	-0.002265	0.0007758	0.7790
100% vs. 90%	0.2065	-0.0002455	-0.001766	0.001275	0.9994
100% vs. 110%	0.2061	0.0001150	-0.001406	0.001635	0.9997
100% vs. 120%	0.2065	-0.0002426	-0.001763	0.001278	0.9994
100% vs. 130%	0.2060	0.0002525	-0.001268	0.001773	0.9993
100% vs. 140%	0.2062	2.210e-006	-0.001518	0.001523	>0.9999
100% vs. 150%	0.2062	-2.253e-005	-0.001543	0.001498	>0.9999

$P_{two\text{-}sided}$ value in bold and italics were shown as statistically different between groups. Each $P_{two\text{-}sided}$ value was adjusted to account for multiple comparisons.

Supplementary Table 7. Statistics in ten folds cross-validation in sCCA analysis.

	Training dataset	Test dataset
**First correlate**	0.204 (9.47×10^{-4})	0.201 (0.009)
**Second correlate**	0.111 (5.90×10^{-4})	0.104 (0.005)
**Third correlate**	0.054 (6.82×10^{-4})	0.037 (0.009)

Data are shown as mean (standard deviation) of canonical correlation r value across ten folds.

Supplementary Table 8. sCCA analysis between urban living environment and psychiatric symptoms in males and females, separately

	Training dataset		Test dataset		
	r	**Two-sided P_{perm}**	***r***	**Two-sided P_{perm}**	**P_{FDR}**
**Females**					
First correlate	0.205	***P<0.001***	0.196	***P<0.001***	***P<0.001***
Second correlate	0.118	***P<0.001***	0.101	***P<0.001***	***P<0.001***
Third correlate	0.059	***P<0.001***	0.043	0.179	0.277
**Males**					
First correlate	0.206	***P<0.001***	0.188	***P<0.001***	***P<0.001***
Second correlate	0.107	***P<0.001***	0.086	***P<0.001***	***P<0.001***
Third correlate	0.062	***P<0.001***	0.035	***P<0.001***	***0.002***

Supplementary Table 9. 3,436 significant GWAS associations between SNPs and affective symptoms group after Bonferroni correction of *Pc*<0.05 (see Excel)

Supplementary Table 10. 29 significant GWAS associations between SNPs and anxiety symptoms group after Bonferroni correction of *Pc*<0.05 (see Excel)

Supplementary Table 11. 10 significant GWAS associations between SNPs and emotional instability symptoms group after Bonferroni correction of *Pc*<0.05 (see Excel)

Supplementary Table 12. Gene-set enrichment analysis in gene ontology of affective, anxiety and emotional instability symptom groups associated genes after Bonferroni correction of *Pc*<0.05 (see Excel)

Supplementary Table 13. Replications of gene scores associated with psychiatric symptom groups in UKB-NI dataset (see Excel)

Supplementary Table 14. Weights for brain volumes simultaneously associated with urban living environment profiles and psychiatric symptoms groups in msCCA-regression.

Mental health	Brain area	Weight
Affective symptom group	Right Frontal Pole	-0.239
	Left Superior Frontal Gyrus	-0.457
	Right Superior Frontal Gyrus	-0.429
	Right Occipital Fusiform Gyrus	-0.242
	Right Crus I Cerebellum	-0.319
	Right Crus II Cerebellum	-0.287
	Right VIIb Cerebellum	-0.332
	Left VIIb Cerebellum	-0.256
	Right VIIIa Cerebellum	-0.438
	Left VIIIa Cerebellum	-0.296
	Right VIIIb Cerebellum	-0.328
	Left Amygdala	-0.278
	Right Ventral Striatum	-0.246
Anxiety symptom group	Left Inferior Frontal Gyrus	-0.250
	Left Juxtapositional Lobule Cortex	-0.287
	Right V Cerebellum	-0.301
	Left VI Cerebellum	-0.278
	Right Crus I Cerebellum	-0.351
	Left Crus I Cerebellum	-0.289
	Left VIIIa Cerebellum	-0.274
	Right VIIIa Cerebellum	-0.396
	Left VIIIb Cerebellum	-0.300
	Right VIIIb Cerebellum	-0.291
	Right Amygdala	-0.277
Emotional instability symptom group	Left Frontal Pole	-0.339

Right Frontal Pole	-0.344
Left Insular Cortex	-0.276
Right Insular Cortex	-0.262
Left Precentral Gyrus	-0.391
Right Precentral Gyrus	-0.321
Left Postcentral Gyrus	-0.268
Left Lateral Occipital Cortex	-0.275
Left Central Opercular Cortex	-0.270
Right Frontal Orbital Cortex	-0.270
Left Amygdala	-0.278
Right Amygdala	-0.292
Left Crus I Cerebellum	-0.269

Supplementary Table 15. Moderated mediation statistics in genes scores, urban environmental profile, brain component and psychiatric symptoms groups after bootstrapping (see Excel)

Members list of the environMENTAL Consortium (sorted by partner no.)

Name	Email	Project partner	Institution
Henrik Walter	henrik.walter@charite.de	1 CHARITE	Dept. of Psychiatry and Psychotherapy, CCM, Charite Universitaetsmedizin Berlin, Germany
Andreas Heinz	andreas.heinz@charite.de	1 CHARITE	Dept. of Psychiatry and Psychotherapy, CCM, Charite Universitaetsmedizin Berlin, Germany
Markus Ralser	markus.ralser@charite.de	1 CHARITE	Dept. of Biochemistry, Charité – Universitätsmedizin Berlin, Berlin, Germany<br>Nuffield Department of Medicine, University of Oxford, UK<br>Max Planck Institute for Molecular Genetics, Berlin, Germany
Sven Twardziok	sven.twardziok@charite.de	1 CHARITE	Berlin Institute of Health at Charité – Universitätsmedizin Berlin, Berlin, Germany
Nilakshi Vaidya	nilakshi.vaidya@charite.de	1 CHARITE	Centre for Population Neuroscience and Stratified Medicine (PONS), Charite Mental Health, Dept. of Psychiatry and Psychotherapy, CCM, Charite Universitätsmedizin Berlin, Germany
Emin Serin	emin.serin@charite.de	1 CHARITE	Centre for Population Neuroscience and Stratified Medicine (PONS), Charite Mental Health, Dept. of Psychiatry and Psychotherapy, CCM, Charite Universitätsmedizin Berlin, Germany
Marcel Jentsch	marcel.jentsch@charite.de	1 CHARITE	Centre for Population Neuroscience and Stratified Medicine (PONS), Dept. of Psychiatry and Psychotherapy, CCM, Charite Universitaetsmedizin Berlin, Germany<br>Berlin Institute of Health at Charité – Universitätsmedizin Berlin, Center of Digital Health, Berlin, Germany
Esther Hitchen	esther.hitchen@charite.de	1 CHARITE	Centre for Population Neuroscience and Stratified Medicine (PONS), Charite Mental Health, Dept. of Psychiatry and Psychotherapy, CCM, Charite Universitätsmedizin Berlin, Germany

Name	Email	Project partner	Institution
Roland Eils	roland.eils@charite.de	1 CHARITE	Berlin Institute of Health at Charité – Universitätsmedizin Berlin, Center of Digital Health, Berlin, Germany
Ulrike-Helene Taron	Ulrike-helene.taron@charite.de	1 CHARITE	Berlin Institute of Health at Charité – Universitätsmedizin Berlin, Center of Digital Health, Berlin, Germany
Tatjana Schütz	tatjana.schuetz@bih-charite.de	1 CHARITE	Berlin Institute of Health at Charité – Universitätsmedizin Berlin, Center of Digital Health, Berlin, Germany
Kerstin Schepanski	kerstin.schepanski@fu-berlin.de	2 FUB	Institute of Meteorology, Free University Berlin, Berlin, Germany
James Richard Banks	James.banks@fu-berlin.de	2 FUB	Institute of Meteorology, Free University Berlin, Berlin, Germany
Tobias Banaschewski	tobias.banaschewski@zi-mannheim.de	3 ZISG	Department of Child and Adolescent Psychiatry and Psychotherapy, Central Institute of Mental Health, Medical Faculty Mannheim, Heidelberg University, Germany
Andreas Meyer-Lindenberg	a.meyer-lindenberg@zi-mannheim.de	3 ZISG	Central Institute of Mental Health, Medical Faculty Mannheim, Heidelberg University, Germany
Tobias Banaschewski	tobias.banaschewski@zi-mannheim.de	3 ZISG	Central Institute of Mental Health, Medical Faculty Mannheim, Heidelberg University, Germany
Heike Tost	heike.tost@zi-mannheim.de	3 ZISG	Central Institute of Mental Health, Medical Faculty Mannheim, Heidelberg University, Germany
Nathalie Holz	nathalie.holz@zi-mannheim.de	3 ZISG	Department of Child and Adolescent Psychiatry and Psychotherapy, Central Institute of Mental Health, Medical Faculty Mannheim / Heidelberg University, Mannheim, Germany<br>Donders Institute for Brain, Cognition and Behavior, Radboud University Nijmegen, Nijmegen, the Netherlands<br>Department for Cognitive Neuroscience, Radboud University Medical Center Nijmegen, Nijmegen, the Netherlands

Name	Email	Project partner	Institution
			Institute of Medical Psychology and Medical Sociology, University Medical Center Schleswig Holstein, Kiel University, Kiel, Germany
Emanuel Schwarz	emanuel.schwarz@zi-mannheim.de	3 ZISG	Central Institute of Mental Health, Medical Faculty Mannheim, Heidelberg University, Germany
Argyris Stringaris	a.stringaris@ucl.ac.uk	3 ZISG	Current Position<br>Professor of Child and Adolescent Psychiatry<br>Division of Psychiatry and Department of Clinical, Educational & Health Psychology University College London<br><br>Other Current Positions<br>Professor of Child and Adolescent Psychiatry<br>University of Athens, Greece<br><br>Visiting Scientist and Co-Head of the Clinical Neuroscience of Mood Disorders in Children and Adolescents<br>Central Institute of Mental Health (Zentralinstitut für Seelische Gesundheit), Mannheim, Germany<br><br>Honorary Consultant Child & Adolescent Psychiatrist<br>Camden & Islington NHS Foundation Trust
Maja Neidhart	maja.neidhart@charite.de<br><br>MajaNadine.Neidhart@zi-mannheim.de	3 ZISG	Dept. of Psychiatry and Psychotherapy, CCM, Charite Universitaetsmedizin Berlin, Germany<br><br>Department of Child and Adolescent Psychiatry and Psychotherapy, Central Institute of Mental Health, Medical Faculty Mannheim, Heidelberg University, Germany

Name	Email	Project partner	Institution
Nina Christmann	Nina.christmann@zi-mannheim.de	3 ZISG	Department of Child and Adolescent Psychiatry and Psychotherapy, Central Institute of Mental Health, Medical Faculty Mannheim, Heidelberg University, Germany
Karina Jansone	Karina.jansone@zi-mannheim.de	3 ZISG	Department of Child and Adolescent Psychiatry and Psychotherapy, Central Institute of Mental Health, Medical Faculty Mannheim, Heidelberg University, Germany
Frauke Nees	nees@med-psych.uni-kiel.de	4 UKSH	Institute of Medical Psychology and Medical Sociology, University Medical Center Schleswig-Holstein, Kiel University, Kiel, Germany
Sebastian Siehl	siehl@med-psych.uni-kiel.de	4 UKSH	Institute of Medical Psychology and Medical Sociology, University Medical Center Schleswig-Holstein, Kiel University, Kiel, Germany
Ole A. Andreassen	ole.andreassen@medisin.uio.no	5 UiO	Norwegian Centre for Mental Disorders Research (NORMENT), Division of Mental Health and Addiction, Oslo University Hospital & Institute of Clinical Medicine, University of Oslo, Norway<br>K.G. Jebsen Centre for Neurodevelopmental Disorders, University of Oslo, Norway
Lars T. Westlye	l.t.westlye@psykologi.uio.no	5 UiO	Department of Psychology, University of Oslo, Norway<br>Norwegian Centre for Mental Disorders Research (NORMENT), Division of Mental Health and Addiction, Oslo University Hospital & Institute of Clinical Medicine, University of Oslo, Norway<br>K.G. Jebsen Centre for Neurodevelopmental Disorders, University of Oslo, Norway
Dennis van der Meer	d.van.der.meer@medisin.uio.no	5 UiO	Norwegian Centre for Mental Disorders Research (NORMENT), Division of Mental Health and Addiction, Oslo University Hospital & Institute of Clinical Medicine, University of Oslo, Norway
Helga Ask	helga.ask@psykologi.uio.no	5 UiO	Department of Psychology, University of Oslo, Norway

Name	Email	Project partner	Institution
			Department of mental disorders, Norwegian Institute of Public Health, Norway
Sara Fernandez	sarafrcabello@gmail.com	5 UiO	Norwegian Centre for Mental Disorders Research (NORMENT), Division of Mental Health and Addiction, Oslo University Hospital & Institute of Clinical Medicine, University of Oslo, Norway
Rikka Kjelkenes	rikkakj@uio.no	5 UiO	Norwegian Centre for Mental Disorders Research (NORMENT), Division of Mental Health and Addiction, Oslo University Hospital & Institute of Clinical Medicine, University of Oslo, Norway
Michael Rapp	michael.rapp@uni-potsdam.de	6 UP	Departmentof Social and Preventive Medicine, University of Potsdam, Potsdam, Germany
Mira Tschorn	mira.tschorn@uni-potsdam.de	6 UP	Department of Social and Preventive Medicine, University of Potsdam, Potsdam, Germany
Sarah Jane Böttger	sarah.boettger@uni-potsdam.de	6 UP	Department of Social and Preventive Medicine, University of Potsdam, Potsdam, Germany
Antoine Bernas	antoine.bernas@donders.ru.nl	7 RUMC	Donders Institute for Brain, Cognition and Behaviour, Radboud University Medical Centre, Nijmegen, the Netherlands
Gaia Novarino	gaia.novarino@ist.ac.at	8 IST AUSTRIA	Institute of Science and Technology, Klosterneuburg, Austria
Lena Marr	lena.marr@ist.ac.at	8 IST AUSTRIA	Institute of Science and Technology, Klosterneuburg, Austria

Name	Email	Project partner	Institution
Mel Slater	melslater@ub.edu	9 UB	Event Lab, Department of Clinical Psychology and Psychobiology, Institute of Neurosciences, University of Barcelona, Casanova, 143, 08036 Barcelona, Spain<br>Department of Computer Science, University College London, London, UK
Guillem Feixas Viapiana	gfeixas@ub.edu	9 UB	Institut de Neurociències, Universitat de Barcelona, Campus de Mundet, Barcelona, Spain
Francisco Eiroa Orosa	feiroa@ub.edu	9 UB	Institut de Neurociències, Universitat de Barcelona, Campus de Mundet, Barcelona, Spain
Jaime Gallego	jgallego@ub.edu	9 UB	Institut de Neurociències, Universitat de Barcelona, Campus de Mundet, Barcelona, Spain
Alvaro Pastor	alvaropastorphd@gmail.com	9 UB	Institut de Neurociències, Universitat de Barcelona, Campus de Mundet, Barcelona, Spain
Markus M. Nöthen	markus.noethen@uni-bonn.de	10 UKB	Institute of Human Genetics, University of Bonn, School of Medicine & University Hospital Bonn, Bonn, Germany
Andreas J. Forstner	forstner@uni-bonn.de	10 UKB	Institute of Human Genetics, University of Bonn, School of Medicine & University Hospital Bonn, Bonn, Germany<br>Institute of Neuroscience and Medicine (INM-1), Research Center Jülich, Germany

Name	Email	Project partner	Institution
Isabelle Claus	isabelle.claus@ukbonn.de	10 UKB	Institute of Human Genetics, University of Bonn, School of Medicine & University Hospital Bonn, Bonn, Germany
Per Hoffmann	phoffmann@lifeandbrain.com	11 LAB	Institute of Human Genetics, University of Bonn, School of Medicine & University Hospital Bonn, Bonn, Germany
Abbi Miller	abigail.miller@uni-bonn.de	11 LAB	Institute of Human Genetics, University of Bonn, School of Medicine & University Hospital Bonn, Bonn, Germany
Stefanie Heilmann-Heimbach	sheilmann@lifeandbrain.com	11 LAB	Institute of Human Genetics, University of Bonn, School of Medicine & University Hospital Bonn, Bonn, Germany
Peter Sommer	peter.sommer@ksilink.com	12 KSILINK	Ksilink, Strasbourg, France
Mona Boye	mona.boye@ksilink.com	12 KSILINK	Ksilink, Strasbourg, France
Johannes Wilbertz	johannes.wilbertz@ksilink.com	12 KSILINK	Ksilink, Strasbourg, France
Karen Schmitt	karen.schmitt@ksilink.com	12 KSILINK	Ksilink, Strasbourg, France
Viktor Jirsa	viktor.jirsa@univ-amu.fr	13 AMU	Aix Marseille Université, Institut National de la Santé et de la Recherche Médicale (Inserm), Institut de Neurosciences des Systèmes (INS) UMR1106, Marseille, France
Spase Petkoski	spase.petkoski@univ-amu.fr	13 AMU	Aix Marseille Université, Institut National de la Santé et de la Recherche Médicale (Inserm), Institut de Neurosciences des Systèmes (INS) UMR1106, Marseille, France

Name	Email	Project partner	Institution
Séverine Pitel	severine.PITEL@univ-amu.fr	13 AMU	Aix Marseille Université, Institut National de la Santé et de la Recherche Médicale (Inserm), Institut de Neurosciences des Systèmes (INS) UMR1106, Marseille, France
Lisa Otten	lisa.otten@univ-amu.fr	13 AMU	Aix Marseille Université, Institut National de la Santé et de la Recherche Médicale (Inserm), Institut de Neurosciences des Systèmes (INS) UMR1106, Marseille, France
Anastasios-Polykarpos Athanasiadis	anastasios-polykarpos.athanasiadis@univ-amu.fr	13 AMU	Aix Marseille Université, Institut National de la Santé et de la Recherche Médicale (Inserm), Institut de Neurosciences des Systèmes (INS) UMR1106, Marseille, France
Charlie Pearmund	c.pearmund@virtualbodyworks.com	14 VBW	Virtual Bodyworks, Barcelona, Spain
Bernhard Spanlang	bspanlang@virtualbodyworks.com	14 VBW	Virtual Bodyworks, Barcelona, Spain
Elena Alvarez	e.alvarez@virtualbodyworks.com	14 VBW	Virtual Bodyworks, Barcelona, Spain
Mavi Sanchez	mavi.sanchez@virtualbodyworks.com	14 VBW	Virtual Bodyworks, Barcelona, Spain
Arantxa Giner	arantxa@virtualbodyworks.com	14 VBW	Virtual Bodyworks, Barcelona, Spain
Sören Hese	soeren.hese@uni-jena.de	16 FSU	Institute of Geography, Friedrich Schiller University Jena, Germany
Paul Renner	paul.renner@uni-jena.de	16 FSU	Institute of Geography, Friedrich Schiller University Jena, Germany
Tianye Jia	tianyejia@fudan.edu.cn	17 FDU	Centre for Population Neuroscience and Stratified Medicine (PONS), Institute for Science and Technology of Brain-inspired Intelligence (ISTBI), Fudan University, Shanghai, China

Name	Email	Project partner	Institution
Yanting Gong	gongyanting9458@163.com	17 FDU	Centre for Population Neuroscience and Stratified Medicine (PONS), Institute for Science and Technology of Brain-inspired Intelligence (ISTBI), Fudan University, Shanghai, China
Yuxiang Dai	18013009209@163.com	17 FDU	Centre for Population Neuroscience and Stratified Medicine (PONS), Institute for Science and Technology of Brain-inspired Intelligence (ISTBI), Fudan University, Shanghai, China
Yunman Xia	xiayunman@outlook.com	17 FDU	Centre for Population Neuroscience and Stratified Medicine (PONS), Institute for Science and Technology of Brain-inspired Intelligence (ISTBI), Fudan University, Shanghai, China
Xiao Chang	xchang@fudan.edu.cn	17 FDU	Centre for Population Neuroscience and Stratified Medicine (PONS), Institute for Science and Technology of Brain-inspired Intelligence (ISTBI), Fudan University, Shanghai, China
Vince Calhoun	vcalhoun@gsu.edu	18 GSURF	Tri-institutional Center for Translational Research in Neuroimaging and Data Science (TReNDS), Georgia State, Georgia Tech, Emory, Atlanta, GA, USA
Jingyu Liu	jliu75@gsu.edu	18 GSURF	Tri-institutional Center for Translational Research in Neuroimaging and Data Science (TReNDS), Georgia State, Georgia Tech, Emory, Atlanta, GA, USA
Paul Thompson	pthomp@usc.edu	19 UNIS.CALIFS	Imaging Genetics Center, Mark & Mary Stevens Institute for Neuroimaging & Informatics, Los Angeles, CA, USA
Nicholas Clinton	nclinton@google.com	20 Google	Google, Inc., Mountain View, CA, USA
Sylvane Desrivières	sylvane.desrivieres@kcl.ac.uk	21 KCL	Social, Genetic and Developmental Psychiatry Centre, Institute of Psychiatry, Psychology & Neuroscience, King’s College London, UK

Name	Email	Project partner	Institution
Allan Young	allan.young@kcl.ac.uk	21 KCL	Institute of Psychiatry, Psychology & Neuroscience, SGDP Centre, King's College London, UK
Bernd Stahl	Bernd.Stahl@nottingham.ac.uk	22 DMU	School of Computer Science, University of Nottingham, UK
George Ogoh	george.ogoh@dmu.ac.uk	22 DMU	School of Computer Science, University of Nottingham, UK
