# Supplementary material for: Effects of urban living environments on mental health in adults
Source: Nat Med. 2023 Jun 15;29(6):1456–67. doi: 10.1038/s41591-023-02365-w (PMC10287556; doi:10.1038/s41591-023-02365-w)
Supplement: Supplementary file 2 — Reporting Summary [file 41591_2023_2365_MOESM2_ESM.pdf]

nature portfolio

Corresponding author(s): Gunter Schumann

Last updated by author(s): Feb 22, 2023

Reporting Summary

Nature Portfolio wishes to improve the reproducibility of the work that we publish. This form provides structure for consistency and transparency in reporting. For further information on Nature Portfolio policies, see our Editorial Policies and the Editorial Policy Checklist.

Statistics

For all statistical analyses, confirm that the following items are present in the figure legend, table legend, main text, or Methods section.

n/a	Confirmed	
☐	☒	The exact sample size (*n*) for each experimental group/condition, given as a discrete number and unit of measurement
☐	☒	A statement on whether measurements were taken from distinct samples or whether the same sample was measured repeatedly
☐	☒	The statistical test(s) used AND whether they are one- or two-sided<br>*Only common tests should be described solely by name; describe more complex techniques in the Methods section.*
☐	☒	A description of all covariates tested
☐	☒	A description of any assumptions or corrections, such as tests of normality and adjustment for multiple comparisons
☐	☒	A full description of the statistical parameters including central tendency (e.g. means) or other basic estimates (e.g. regression coefficient) AND variation (e.g. standard deviation) or associated estimates of uncertainty (e.g. confidence intervals)
☐	☒	For null hypothesis testing, the test statistic (e.g. *F*, *t*, *r*) with confidence intervals, effect sizes, degrees of freedom and *P* value noted<br>*Give P values as exact values whenever suitable.*
☒	☐	For Bayesian analysis, information on the choice of priors and Markov chain Monte Carlo settings
☒	☐	For hierarchical and complex designs, identification of the appropriate level for tests and full reporting of outcomes
☐	☒	Estimates of effect sizes (e.g. Cohen's *d*, Pearson's *r*), indicating how they were calculated

Our web collection on statistics for biologists contains articles on many of the points above.

Software and code

Policy information about availability of computer code

Data collection	Data availability<br>The UK Biobank data used in the study are all available in the https://www.ukbiobank.ac.uk. The Human Protein Atlas portal was available in https://www.proteinatlas.org/.<br>Code availability<br>The core code used to run the analyses reported in this study can be found at: https://github.com/jiayuanqqxu/scca-regression.
Data analysis	1. R package lavaan v0.6-12, R package caret v6.0-92 and R package mixoimcs v6.22.0 was used to analysis multivariate relationship between urban environment, brain and mental health data.<br>2. Plink 2.0 and BGENIE v1.2 was used to preprocess imputed genetic data and GWAS analysis of symptom groups of mental illness.<br>3. ToppGene portal (https://toppgene.cchmc.org/) and FUMA portal (https://fuma.ctglab.nl/) was used to identify significant enrichments for tissue-specificity and gene ontology (GO).<br>4. R package process v3.5beta was used test the multivariate association between the urban environmental profile, gene scores, brain volume measures and psychiatric symptom groups.

For manuscripts utilizing custom algorithms or software that are central to the research but not yet described in published literature, software must be made available to editors and reviewers. We strongly encourage code deposition in a community repository (e.g. GitHub). See the Nature Portfolio guidelines for submitting code & software for further information.

Data

Policy information about availability of data

All manuscripts must include a data availability statement. This statement should provide the following information, where applicable:

- Accession codes, unique identifiers, or web links for publicly available datasets
- A description of any restrictions on data availability
- For clinical datasets or third party data, please ensure that the statement adheres to our policy

The study used the following data from UK Biobank dataset:

1. Urban environment data: Urban Morphometric Platform and UK Biobank related local environment data was used to assess the social and physical environment data (air and sound pollution, traffic, greenspace proximity, coastal and water proximity, socioeconomic indices of multiple deprivation, building class, destination accessibility, land use density, terrain, NDVI and street network accessibility) based on the subjects' geolocation.

2. Mental health data: There are 44 mental health items in the category 'Mental health' in the UK Biobank that cover symptoms of affective and anxiety disorders, as well as personality (category id:100060). These items were obtained from a standardised mental health questionnaire that participants answered at the time of recruitment. Of this questionnaire 21 items were excluded because the missing rate was larger than 50% from 502,616 participants of UK Biobank.

3. Genetic data: We used the imputed genomic data (Version 3) made available by UK Biobank with 487,411 individuals, which was imputed from the Haplotype Reference Consortium (HRC) reference panel and a merged UK10K and 1000 Genomes phase 3 reference panels.

4. Neuroimaging data: T1-weighted imaging were acquired from one 3.0-Tesla MRI scanner from Siemens® Skyra running VD13A SP4 with a standard 32-channel radiofrequency receive head coil at UK Biobank imaging center in Cheadle Manchester.

Human research participants

Policy information about studies involving human research participants and Sex and Gender in Research.

Reporting on sex and gender

Gender were considered in the study design and included as a covariate where relevant. Also the sCCA analysis between urban living environment and symptoms of mental illness was also applied in males and females, respectively.

Population characteristics

1. CFA and sCCA between urban living environment and psychiatric symptoms:
   n= 141,087
   age: 59.25(8.14)
   male/female:65,505/75,582
2. GWAS of environmental psychiatric symptom-group
   n=76,508
   age=59.55 (8.02)
   male/female: 36,557/39,951
3. msCCA between urban living environment profile, brain volume and psychiatric symptom-group:
   n=14,988
   age=57.77 (7.49)
   male/female:7,265/7,723
4. Moderated mediation analysis
   n=8,705
   age=58.06 (7.42)
   male/female:4,278/4,427

Recruitment

The UK Biobank (UKBB) is a population-based cohort including 502,616 participants recruited in the United Kingdom between 2006 and 2010. Participants who were registered with the National Health Service and living within a 40 km radius of one of the 22 assessment centres in England, Wales, and Scotland were invited to enter the cohort. Among the 502,616 participants, the mean age at baseline was 56.53 years (standard deviation 8.10), 54.41% were men and 81.51% were of White ethnicity. The average Townsend Deprivation Index, a measure of regional socioeconomic status, was -1.29 with a standard deviation of 3.09, thus showing slightly less deprivation than the UK average (scaled at 0). And 11.49%-56.51% participants had ever suffered from different mental symptoms at baseline. The main goals of UKBB are to explore the aetiology of common complex diseases by investigation of their association with underlying genetic and lifestyle determinants, which may contributor to the advancement of modern medicine and treatment that improve human health. Baseline assessments included genomics, physical and social exposures, sociodemographic, lifestyle, occupational, psychosocial and environmental measures. Informed consent was obtained from all UKBB participants.

Ethics oversight

This study was covered by the ethical approval from UKBB, which has been granted by the National Information Governance Board for Health and Social Care and the NHS North West Multicenter Research Ethics Committee. All participants provided informed consent through electronic signature at baseline assessment. The data collected at baseline was used in this study.

Note that full information on the approval of the study protocol must also be provided in the manuscript.

Field-specific reporting

Please select the one below that is the best fit for your research. If you are not sure, read the appropriate sections before making your selection.

☒ Life sciences ☐ Behavioural & social sciences ☐ Ecological, evolutionary & environmental sciences

For a reference copy of the document with all sections, see nature.com/documents/nr-reporting-summary-flat.pdf

Life sciences study design

All studies must disclose on these points even when the disclosure is negative.

Sample size	1. The participants from UK Biobank with complete urban-living environmental variables and psychiatric symptoms data (n=156,075) were divided into datasets without neuroimaging data (n=141,087, UKB-nonNI) and with neuroimaging data (n=14,988, UKB-NI). To avoid overestimating the variance shared between urban-living environment categories and psychiatric symptoms, we used a split-data analysis design with a training dataset of 90% of the data (n=126,978), and a test dataset of 10% (n=14,109) in the 141,087 participants of the UKB-nonNI dataset.<br>2. We performed GWAS analyses of the canonical variates of the affective, anxiety and emotional instability symptom-groups, respectively, in 76,508 participants with complete genetic, urban-environment and psychiatric symptoms in UKB-nonNI datasets.<br>3. To investigate the neurobiology underlying the urban-living environment and psychiatric symptoms , we carried out msCCA on the urban-living environment profiles, regional brain-volume and psychiatric symptom groupss. This analysis was conducted in an independent UKB-NI dataset (n=14,988), split in a into training (90%) and test datasets (10%).<br>4. To test whether the relationships of urban-living environment profiles with psychiatric symptom groups were mediated by brain-volume and moderated by genetic differences, we independently performed moderated-mediation analysis for each replicated gene scores (moderating variable), three urban-living environment profiles (independent variable), three brain-volume components (mediated variable) and three psychiatric symptom groups (dependent variable) in 8,705 adult participants with complete data.
Data exclusions	1. In the sCCA between urban living environment and mental health symptom, only the 156,075 participants with complete 128 urban living environmental data and 21 mental health data were included. These participants were divided into datasets without neuroimaging data (n= 141,087, UKB-nonNI) and with neuroimaging data (n=14,988, UKB-NI).<br>2. In the GWAS of symptom groups of mental illness , only the 76,508 participants with complete genomic, environment and mental health data from the UKB-nonNI dataset were included.<br>3. In the 14,988 participants with environment, brain volume and mental health data in UKB-NI, we applied the mSCCA to test relations between the environmental profiles, regional brain volume and the emotional symptom.<br>4. In the 8,705 participants with complete genomic, environment, brain volume and mental health data in UKB-NI, we then investigated the mediation of the effect of the environmental profiles on emotional symptom groups by regional brain volume and its genetic moderation.
Replication	To replicate the multivariate relation between urban-living environment and mental health, we applied the sCCA analysis in an independent dataset of 14,988 participants with complete environmental, mental health and neuroimaging data from UKB-NI dataset. Again, we used a training dataset (n=13,490, 90%) and a test dataset (n=1,498, 10%), a re-sampling method to ensure variable stability (with a threshold of 90% for non-zero weights from re-sampled data to consider as stable variables) and permutation tests to assess the significance of the results (10,000 times) as we used in the discovery sCCA analysis. Next, we independently replicated the significant SNPs associated with symptom-groups of mental illness surviving from the discovery GWAS analysis (UKB-nonNI dataset) in an independent 8,705 participants of UKB-NI dataset at Bonferroni Pc<0.05 (uncorrected P<0.05/the numbers of all significant SNPs of GWAS of symptoms groups of mental illness from discovery analysis). Then we calculated the corresponding genes scores as the same way we did in the discovery analysis. And finally, we independently validated the associations between genes scores and symptoms group of mental illness in the UKB-NI dataset.
Randomization	We don't have group allocation.
Blinding	We don't have group allocation

Reporting for specific materials, systems and methods

We require information from authors about some types of materials, experimental systems and methods used in many studies. Here, indicate whether each material, system or method listed is relevant to your study. If you are not sure if a list item applies to your research, read the appropriate section before selecting a response.

Materials & experimental systems

n/a	Involved in the study
☒	☐ Antibodies
☒	☐ Eukaryotic cell lines
☒	☐ Palaeontology and archaeology
☒	☐ Animals and other organisms
☒	☐ Clinical data
☒	☐ Dual use research of concern

Methods

n/a	Involved in the study
☒	☐ ChIP-seq
☒	☐ Flow cytometry
☐	☒ MRI-based neuroimaging

Magnetic resonance imaging

Experimental design

Field	Response
Design type	No task fMRI was involved in this work. Only T1-weighted imaging were acquired from one 3.0-Tesla MRI scanner from Siemens® Skyra running VD13A SP4 with a standard 32-channel radiofrequency receive head coil at UK Biobank imaging center in Cheadle Manchester.The standard parameters of a 3D MPRAGE sequence are shown in https://biobank.ctsu.ox.ac.uk/crystal/crystal/docs/brain_mri.pdf.
Design specifications	The standard parameters of a 3D MPRAGE sequence are shown in https://biobank.ctsu.ox.ac.uk/crystal/crystal/docs/brain_mri.pdf.
Behavioral performance measures	There are 44 mental health items in the category 'Mental health' in the UK Biobank that cover symptoms of affective and anxiety disorders, as well as personality (category id:100060). These items were obtained from a standardised mental health questionnaire that participants answered at the time of recruitment. Of this questionnaire 21 items were excluded because the missing rate was larger than 50% from 502,616 participants of UK Biobank.

Acquisition

Field	Response
Imaging type(s)	T1-weighted structural imaging
Field strength	3.0-Tesla MRI scanner from Siemens® Skyra
Sequence & imaging parameters	T1-weighted structural imaging<br>Resolution: 1x1x1 mm Field-of-view: 208x256x256 matrix Duration: 5 minutes 3D MPRAGE, sagittal, in-plane acceleration iPAT=2, prescan-normalise<br>The superior-inferior field-of-view is large (256mm), at little cost, in order to include reasonable amounts of neck/mouth, as those areas will be of interest to some researchers.
Area of acquisition	A whole brain scan

Diffusion MRI ☐ Used ☒ Not used

Preprocessing

Field	Response
Preprocessing software	BET (Brain Extraction Tool) and FLIRT (FMRIB's Linear Image Regsitration Tool)
Normalization	nonlinear
Normalization template	Montreal Neurological Institute (MNI) 152 space
Noise and artifact removal	correct for variance in individual brain sizes
Volume censoring	NA

Statistical modeling & inference

Field	Response
Model type and settings	1. A total of 53 urban living environment catgories including 128 items were included in this study. In the 53 categories, there were 34 categories having one independent item. In the remaining 19 categories, given that some items of each category examined similar aspects of urban environment. CFA was performed to collapse the urban environment information into 19 latent environmental categories.<br>2. We apply a training and test data split design to investigate the relation of urban living environment with symptoms of mental illness using sparse canonical correlation analysis (sCCA), a multivariate analysis technique to determine multivariate associations between two or more sets of variables.<br>3. To test the underlying genetic information of mental illness which related to environment, GWAS of mental illness symptom group was performed.<br>4. Using a multi-view sparse CCA (msCCA), we identify the regional brain volumes jointly associated with environment profile and mental illness symptom group.<br>5. Using a moderated mediation analysis, we then investigated the mediation of the effect of the environmental profiles on emotional symptom groups by regional brain volume and its genetic moderation
Effect(s) tested	1. Comparative Fit Index , Akaike, sample-size adjusted Bayesian, root mean square error of approximation , standard root mean square residual and Tucker-Lewis index in CFA;<br>2. Canonical correlations coefficient, weight, cross-loading, explained variance in sCCA and msCCA;<br>3. 95% confidence intervals and explained mediation effect in moderated mediation analysis.

Specify type of analysis: ☐ Whole brain ☒ ROI-based ☐ Both

Anatomical location(s)	Neuroimaging data were acquired from one 3.0-Tesla MRI scanner from Siemens® Skyra running VD13A SP4 with a standard 32-channel radiofrequency receive head coil at UK Biobank imaging center in Cheadle Manchester. The standard parameters of a 3D MPRAGE sequence are shown in https://biobank.ctsu.ox.ac.uk/crystal/crystal/docs/brain_mri.pdf. The FAST grey matter segmentation is used to generate a further 139 regional image-derived phenotypes (IDPs), by summing the grey matter partial volume estimates within 139 ROIs: 111 cortical and subcortical gray matter volume (GMV) and 28 cerebellum GMV (field ID 1101). These ROIs are defined in MNI152 space, combining parcellations from several atlases: the Harvard-Oxford cortical and subcortical atlases https://fsl.fmrib.ox.ac.uk/fsl/fslwiki/Atlases and the Diedrichsen cerebellar atlas http://www.diedrichsenlab.org/imaging/propatlas.htm. The detailed information is shown in https://biobank.ctsu.ox.ac.uk/crystal/crystal/docs/brain_mri.pdf.
Statistic type for inference (See Eklund et al. 2016)	NA
Correction	Permutation test, bootstrap, False Discovery Rate correction and Bonferroni correction.

Models & analysis

n/a	Involved in the study
☒	☐ Functional and/or effective connectivity
☒	☐ Graph analysis
☒	☐ Multivariate modeling or predictive analysis
